# A Journey from June 2018 to October 2021 with *N*,*N*-Dimethylformamide and *N*,*N*-Dimethylacetamide as Reactants

**DOI:** 10.3390/molecules26216374

**Published:** 2021-10-21

**Authors:** Jacques Muzart

**Affiliations:** Institut de Chimie Moléculaire de Reims, CNRS—Université de Reims Champagne-Ardenne, B.P. 1039, CEDEX 2, 51687 Reims, France; jacques.muzart@univ-reims.fr

**Keywords:** *N*,*N*-dimethylformamide, DMF, *N*,*N*-dimethylacetamide, DMAc, amination, amidation, formylation, cyanation, insertion, cyclization

## Abstract

A rich array of reactions occur using *N*,*N*-dimethylformamide (DMF) or *N*,*N*-dimethylacetamide (DMAc) as reactants, these two amides being able to deliver their own H, C, N, and O atoms for the synthesis of a variety of compounds. This account highlights the literature published since June 2018, completing previous reviews by the author.

## 1. Introduction

In 2009, I wrote a review highlighting that *N*,*N*-dimethylformamide (DMF) is much more than a solvent of organic and organometallic chemistry [1]. A few years later, I was successively solicited for a book chapter and a review devoted to the use of DMF and *N*,*N*-dimethylacetamide (DMAc) as reagents in synthesis, which have been written with J. Le Bras [2,3]. Reviews related to the DM (DM = DMF or DMAc) reactivity have been published by others [4,5,6,7].

**Scheme 1:**
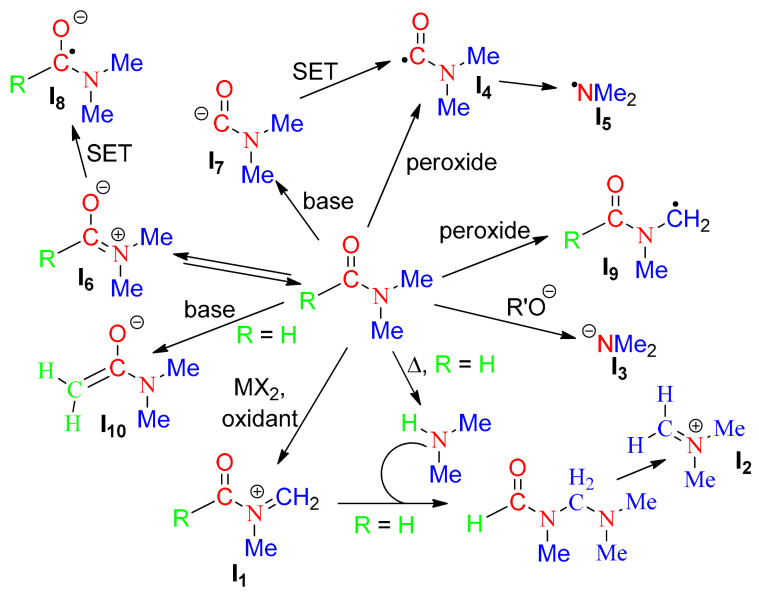
Plausible key species implicated in the delivery of DM fragments (R = H or Me).

The purpose is now to highlight the recent literature, focusing on atom transfer from DM to substrates. A few older reports, useful to place the subject in the appropriate context, or omitted in the previous reviews are included. The processes which necessitate the prerequisite synthesis of DM derivatives such as the Vilsmeier–Haack reagents [8,9] and DMF dimethyl acetal [10] are discarded. Color equations, based on literature proposals, allow easily visualizing the DM atom(s) origin. DM may act as a nucleophilic or electrophilic reagent; neutral, ionic, and radical species, such as those depicted in Scheme 1, may be key intermediates. The reaction mechanisms will be, at best, briefly commented on.

## 2. C Fragment

**Scheme 2:**
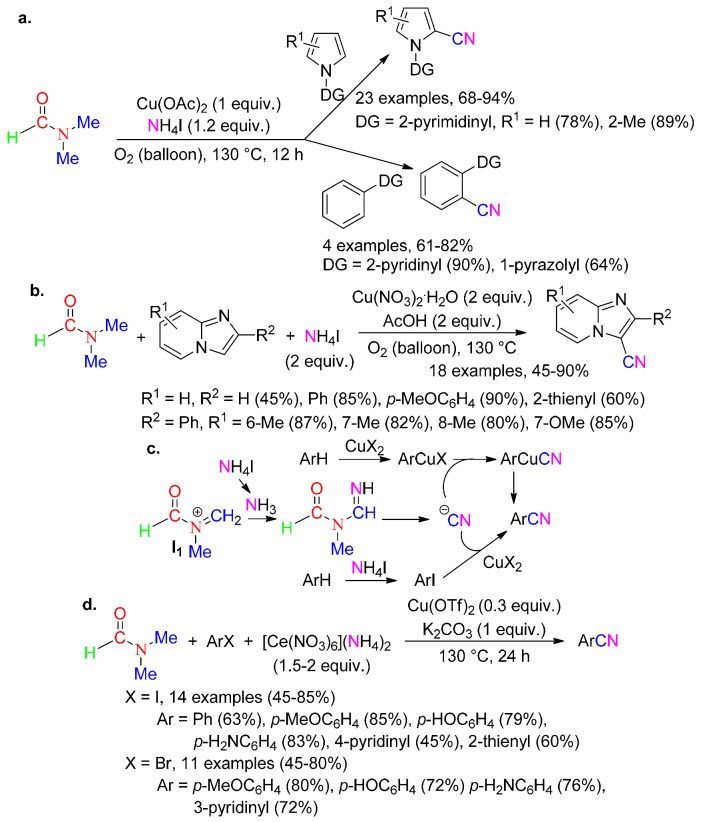
Cyanation of (hetero)arenes and (hetero)aryl halides.

Under aerobic conditions, Cu^II^ mediated the cyanation of (hetero)arenes by the combination of DMF and ammonium iodide (Scheme 2). Both Sun/Cheng’s [11] and Jiang/Ji’s [12] teams assumed a mechanism involving decomposition of NH_4_I, into NH_3_ and production of CN^⊖^ from the addition of NH_3_ to **I_1_** (Scheme 2c). The first team proposed the formation of ArCuCN from ArH and Cu^II^ followed by reductive elimination, while the second team suggested the iodination of ArH and subsequent Cu^II^-mediated cyanation. A recyclable catalyst, obtained from the impregnation of Cu(NO_3_)·3H_2_O over mesoporous siliceous SBA-15 followed by calcination at 450 °C, was used for cyanation of *N*-substituted indoles with NH_4_I and DMF [13]. The NH_4_I/DMF association has also been used for syntheses, as reported in Chinese patents [14,15,16,17,18].

Cu^II^-catalyzed cyanation of (hetero)aryl halides arose using ceric ammonium nitrate and K_2_CO_3_ in DMF (Scheme 2d) [19]. According to Bora and co-workers, K_2_CO_3_ reacts with [Ce(NO_3_)_6_](NH_4_)_2_ leading to (NH_4_)_2_CO_3_ which undergoes decomposition providing NH_3_. The Cu^II^-catalyzed reaction of the latter with DMF affords Cu^I^ and CN^⊖^. The reaction of CN^⊖^ with ArCuX proceeds as disclosed in Scheme 2c. Regeneration of the catalyst is assumed with ceric ammonium nitrate.

## 3. CH Fragment

**Scheme 3:**
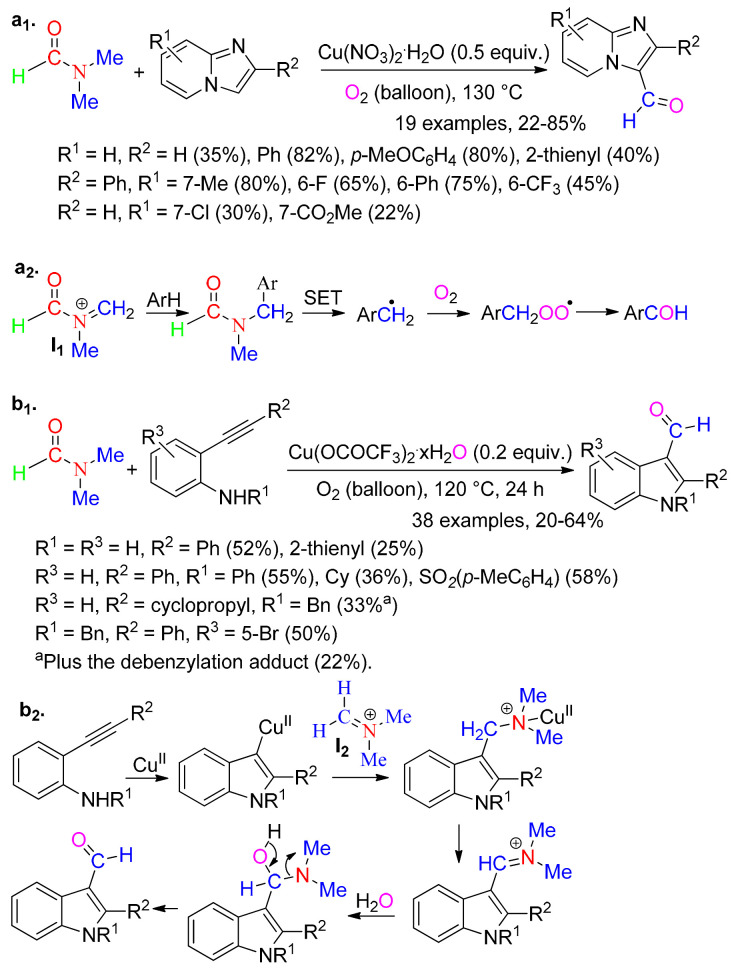
Formylations involving Me.

When carried out in the absence of ammonium iodide, the above Cu-mediated reaction of imidazo [1,2-*a*]pyridines led to the formylation of the C3–H bond (Scheme 3(a_1_)) [12]. The Jiang/Ji team suggested the nucleophilic addition of the substrate to **I_1_** followed by homolytic cleavage of the C–N bond (Scheme 3(a_2_)). Trapping the radical species with oxygen affords a peroxy radical, the decomposition of which gives the product.

According to Lin and co-workers and a reaction with ^18^O-labeled water, the annulative formylation of *o*-alkynylanilines depicted in Scheme 3(b_1_) occurs via activation of the triple bond by coordination to Cu^II^, favoring 5-*endo*-dig cyclization of the substrate leading to a Cu complex which inserts into the C=N bond of I_2_ [20]. The subsequent reductive β-H elimination affords an iminium ion that reacts with water to deliver the product (Scheme 3(b_2_)).

**Scheme 4:**
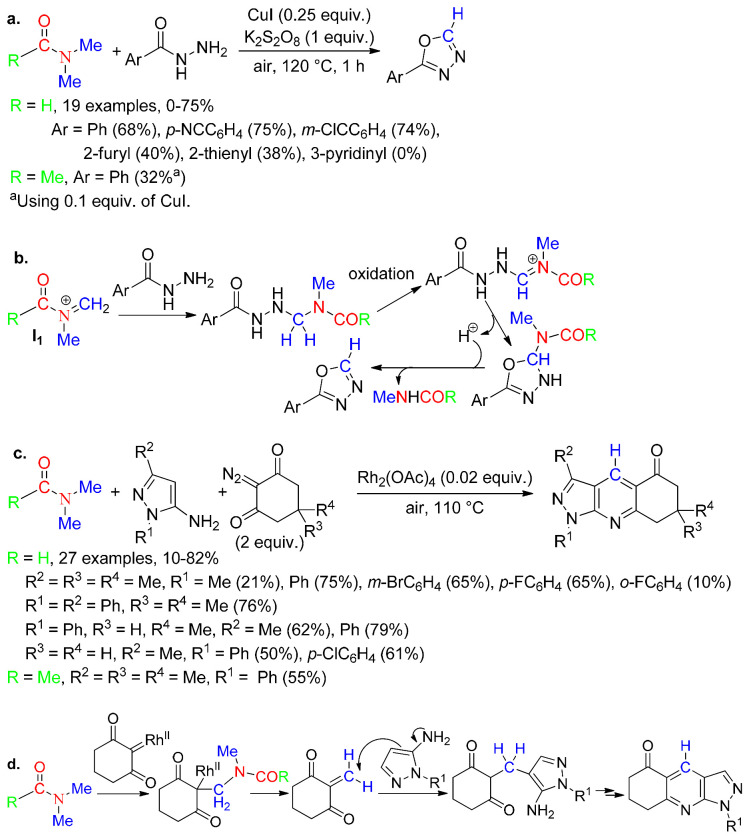

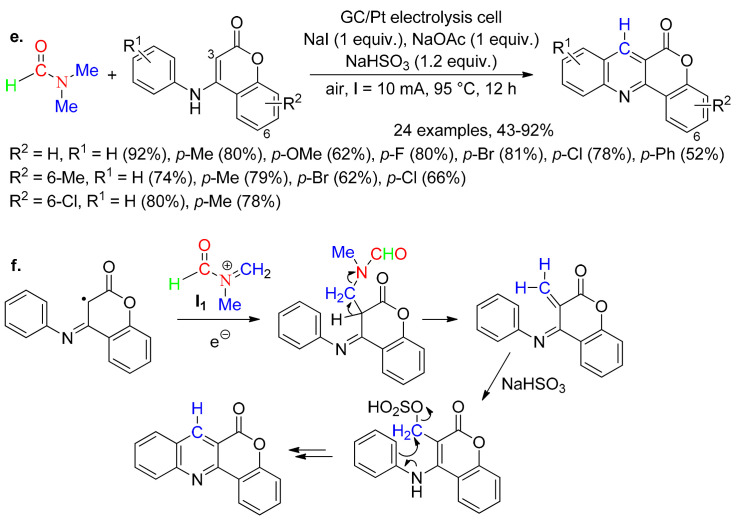
Annulation with methine insertion.

The S. Zhang/Jiang/Jia [21], Xie/Wang/Shang [22], and Weng/Ackermann [23] teams reported annulations with the insertion of a methine fragment (Scheme 4). The addition of hydrazide to **I_1_** is the key step to produce the 1,3,4-oxadiazole (Scheme 4a,b) [21] while the synthesis of the pyrazolo[3,4-*b*]pyridines derivatives (Scheme 4c) [22] is, in fact, due to a Friedel–Crafts-type nucleophilic addition of pyrazol-5-amine to the 2- methylenecyclohexane-1,3-dione (Scheme 4d). The latter is formed by coordination of diazo compound to the Rh catalyst leading to a rhodium-carbene which inserts into a C–H bond of DM, subsequent β-nitrogen elimination delivering the methylene dione and RCONHMe. The recent cyclization of 4-arylaminocoumarins (Scheme 4e) [23] involves the electrochemically NaI-mediated formation of **I_1_** and a radical derivative of the substrate which react together afford the coumarin derivative substituted with CH_2_NMeCOH (Scheme 4f). Elimination of HCONHMe and subsequent NaHSO_3_-mediating cyclization followed by aromatization provide 6*H*-chromeno[4,3-*b*]quinolin-6-one.

**Scheme 5:**
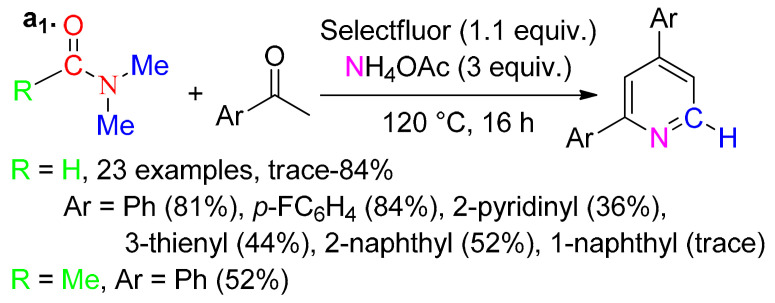

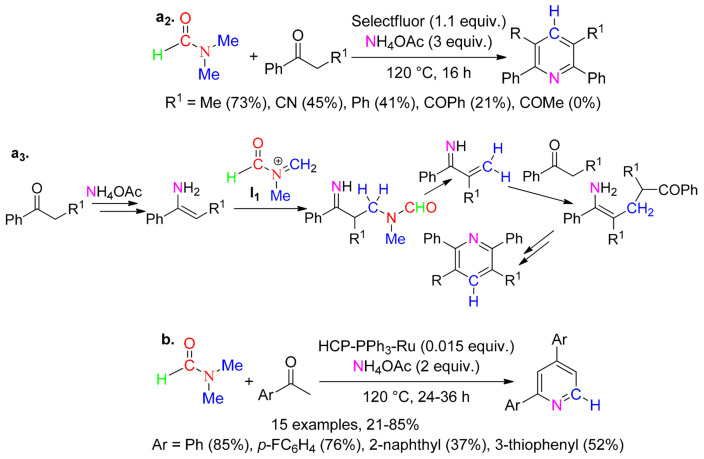
Annulation with insertion of both nitrogen and methane.

An annulation reaction involving the insertion of a nitrogen atom and a CH fragment has been reported from the heating of aromatic ketones (ArCOCH_2_R^1^) in DMF or DMAc in the presence of ammonium acetate and Selectfluor (Scheme 5(a_1_,a_2_)) [24]. The key step leading to symmetrical pyridines (Scheme 5(a_2_)) is the nucleophilic addition of the vinylamine ArC(NH*_2_*)=CHR^1^ formed from the substrate and ammonium acetate, to **I_1_** formed from DMF and Selectfluor (Scheme 5(a_3_)). The resulting intermediate Ar(C=NH)CHR^1^-CH_2_NMeCOH undergoes elimination of HNMeCOH giving Ar(C=NH)CR^1^=CH_2_. A nucleophilic attack of the latter by ArCOCH_2_R^1^ is followed by annulation and aromatization. The unsymmetrical pyridines (Scheme 5(a_1_)) are formed via a rather similar mechanism which starts with homo-condensation of the substrate. The synthesis of 2,4-diphenylpyridine from acetophenone, NH_4_Oac, and DMF or DMAc using NH_4_I instead of Selectfluor was previously reported but in no more than 6% yield [25]. In contrast, the synthesis of such compounds was efficiently catalyzed using RuCl_3_ [3,26] or a recyclable hypercrosslinked polymer-immobilized ruthenium catalyst noted as HCP–PPh_3_–Ru (Scheme 5b) [27].

## 4. CH_2_ Fragment

**Scheme 6:**
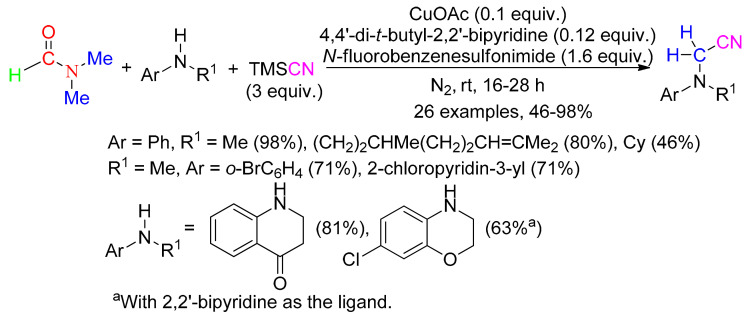
Substitution of secondary aromatic amines with the CH_2_CN moiety.

Copper acetate associated with *N*-fluorobenzenesulfonimide promoted an efficient one-pot three-component condensation leading to α-amino nitriles (Scheme 6) [28]. The reaction involves the addition of a secondary aromatic amine to **I_1_**, giving ArNR^1^CH_2_NMeCOH which is converted into ArNR^1^=CH_2_^⊕^ via cleavage of the CH_2_–NMe bond. Subsequent reaction of the iminium ion with TMSCN provides the α-amino nitrile.

## 5. NC Fragment

To the best of our knowledge, no new report concerned cyanation reactions using the NC fragment of DM.

## 6. NMe_2_ Fragment

### 6.1. Aryl Halides and Tosylates

**Scheme 7:**
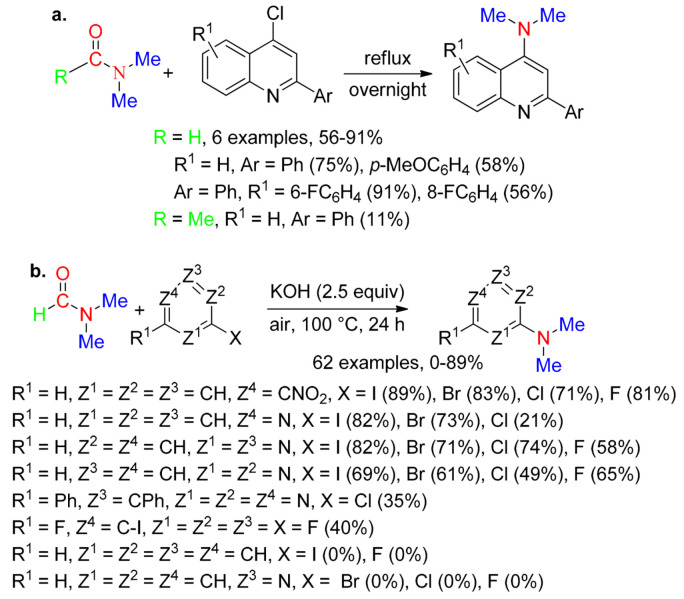

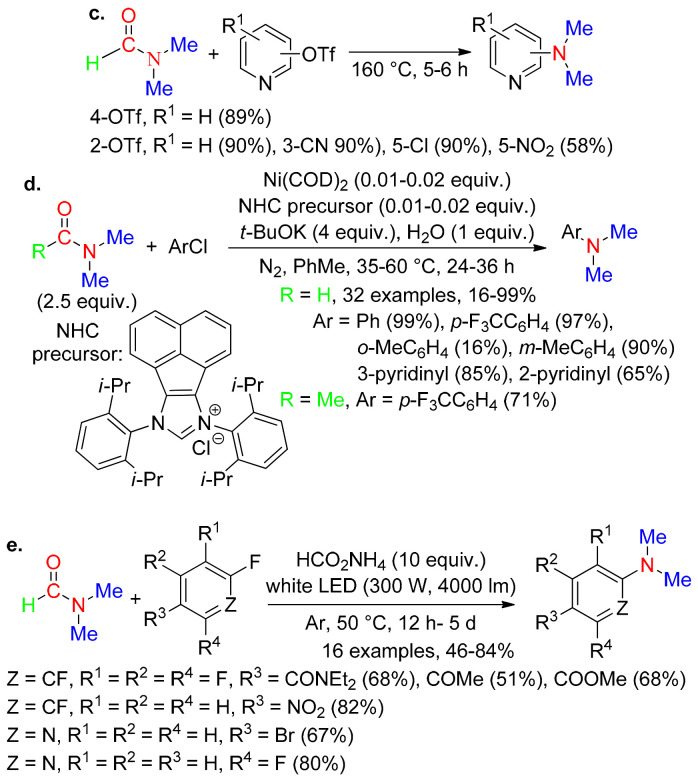
Amination of (hetero)aryl halides and tosylates.

The amination of (hetero)aryl halides or tosylates with DM has been carried out under various conditions (Scheme 7) [29,30,31,32]. With DMF, these reactions could occur via reaction of the substrate with HNMe_2_ produced from thermal or catalytic decomposition of DMF. Under basic conditions, an aromatic nucleophilic substitution (S_N_Ar) process has been discarded and Gong’s team pointed out that the mechanism is unclear [30]. Under the experimental conditions of Scheme 7d, Ni-catalyzed-activation of the C–N bond of DM could participate in the process [33,34], but the presence of water could disfavor the coordination of DM to the transition metal [35]. Cleavage of the C–N bond of amides may, however, arise under transition-metal-free conditions [36].

Recently, the Kozlowski/Handa team disclosed the dimethylamination of fluoro(hetero)arenes with DMF in the presence of ammonium formate and light (Scheme 7e) [37]. According to computational studies and control experiments, the Van der Waals complex formed from the electron-deficient fluorinated aromatic ring and DMF evolves under light toward a charge transfer complex stabilized by ammonium formate. The subsequent decomposition leads to ArNMe_2_ or ArF and HNMe_2_. The S_N_Ar reaction between the two latter species could also contribute to the product formation [37]. Under the optimum conditions, switching the solvent of the reaction of octafluoronaphthalene from DMF to DMAc decreased the yield of 1,3,4,5,6,7,8-heptafluoro-*N*,*N*-dimethylnaphthalen-2-amine from 80% to traces.

### 6.2. Benzyl Ammoniums and Organochlorides

**Scheme 8:**
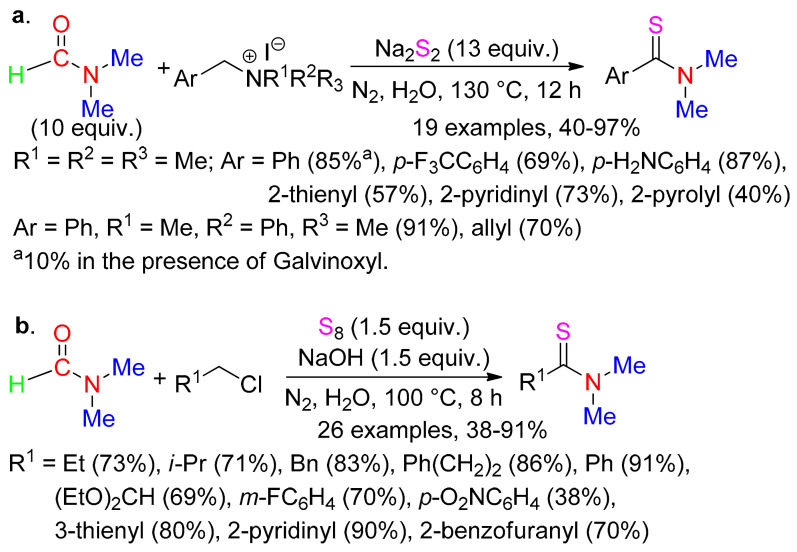
Thioamidation of benzyl ammoniums and organochlorides.

Various *N*,*N*-dimethyl thioamides have been synthetized under aqueous conditions from DMF and quaternary (hetero)benzyl ammonium iodides (Scheme 8a) [38] or primary (hetero)benzyl/alkyl chlorides (Scheme 8b) [39] using sodium disulfide or elemental sulfur and NaOH, respectively. The mechanism of these reactions is unclear. *N*,*N*-Dimethylbenzothioamide was not detected using *N*,*N*,*N*-dimethyl benzylamine instead of benzyl *N*,*N*,*N*-trimethyl ammonium iodide, and isolated in only 10% yield from the ammonium salt in the presence of a radical scavenger (Scheme 8a). According to Cheng’s team, the reaction leading to ArC=SNR_3_ involves radical cross-coupling between ArC^∙^HNR_2_ and Me_2_N^∙^ leading to ArCH(NR_2_)(NMe_2_) which evolves toward the corresponding imminium before undergoing addition of sulfur species, while Ge, Zhou, and co-workers, on the basis of controlling experiments and quantum chemical calculations, assumed a thioamidation of R^1^CH_2_Cl arising via the base-mediated formation of R^1^C^⊖^HCl followed by addition to a S_7_ cluster and then reaction with DMF.

### 6.3. Carbonylated Compounds

**Scheme 9:**
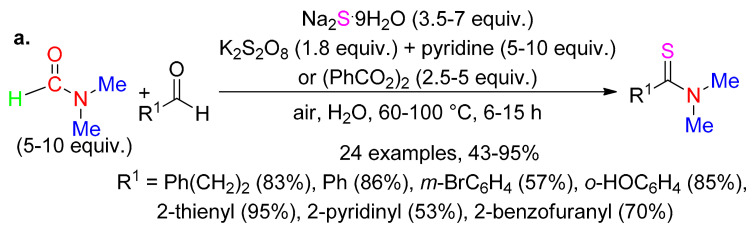

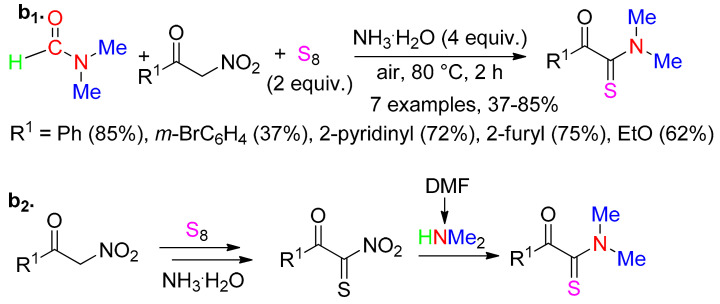
Thioamidation.

Jiang and co-workers reported a procedure also leading to thioamides, but based on the use of alkyl or aryl aldehydes, DMF, and sodium sulfide under aqueous oxidation conditions (Scheme 9a) [40]. According to the proposed mechanism, aqueous sodium sulfide mediates the cleavage of C–N bond providing HNMe_2_ and H_2_S. The addition of sulfur anion to R^1^CH=N^⊕^Me_2_ formed from the condensation of R^1^CHO with HNMe_2,_ followed by oxidation leading to the thioamide.

Denitration of nitroketones mediated with sulfur and aqueous trimethylamine in DMF provided α-ketothioamides (Scheme 9(b_1_)) [41]. Zhang and co-workers proposed that the ketothiolation of the substrate is followed by nucleophilic attack of dimethylamine produced from decarbonylation of DMF (Scheme 9(b_2_)).

**Scheme 10:**
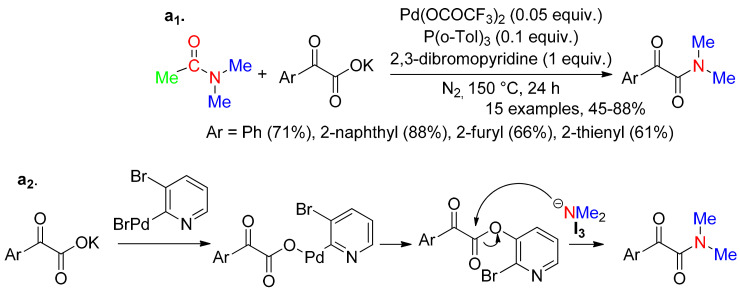
Amidation of arylglyoxylates.

Halopyridines, especially 2,3-dibromopyridine, promote the Pd-catalyzed amidation of arylglyoxylates with DMAc (Scheme 10(a_1_)) [42]. The reaction occurs via Pd-catalyzed esterification of the carboxylate with the halopyridine followed by amidation of the resulting ester with **I_3_** (Scheme 10(a_2_)), the latter being issued from the addition of the in situ formed 2-bromopyridin-3-olate to DMAc.

**Scheme 11:**
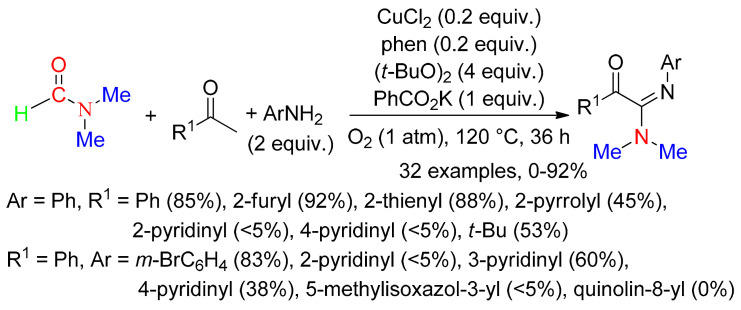
Amidination of methyl ketones.

The Liu/Guo team disclosed the synthesis of 2-oxo-acetamidines from a mixture of methyl ketones and anilines in DMF containing peroxide, base, and Cu^II^ catalyst under oxygen atmosphere (Scheme 11) [43]. According to control experiments, R^1^COCH_2_NMe_2_ produced from radical pathways involving the carbamoyl radical **I_4_** and the aminyl radical **I_5_**, is an intermediate of the reaction.

**Scheme 12:**
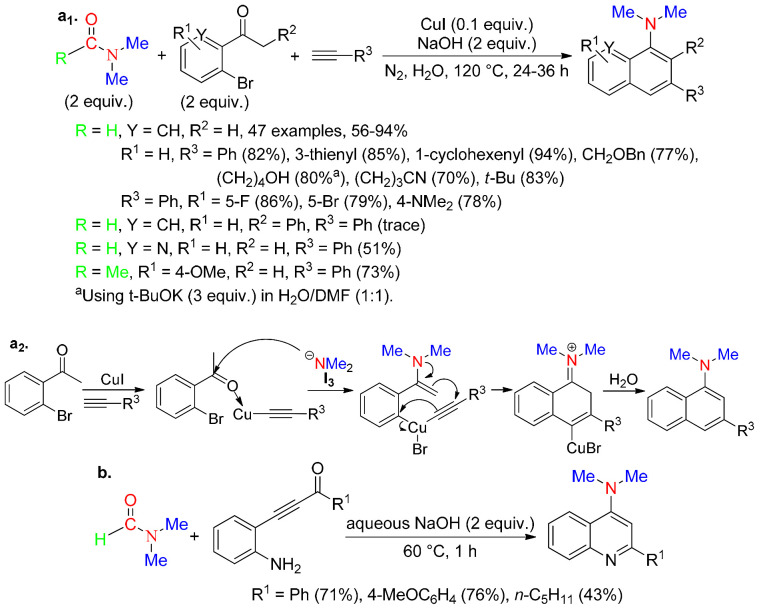
Benzannulations.

A Cu^I^-catalyzed benzannulation leading to functionalized *N*,*N*-dimethylnaphthalen-1-amines or *N*,*N*-dimethylquinolin-8-amine was reported under basic conditions by the Yuan/Zhou team using DM, bromo-(hetero)aryl ketones, and terminal alkynes in water (Scheme 12(a_1_)) [44]. One of the key steps would be the addition of **I_3_** to the alkynylCu-coordinated carbonyl of the ketone (Scheme 12(a_2_)).

*N*,*N*-Dimethylquinolineamines have also been obtained from β-(2-aminophenyl)-α,β-ynones, DMF, and aqueous sodium hydroxide (Scheme 12b) [45].

*N*,*N*-dimethylbenzamide has been formed as a side-product of the base-promoted dehalogenation of aryl halides with PhCHO/DMF [46,47], or in 22% yield from the treatment of benzaldehyde with *t*-BuOK at 90 °C in DMF [46].

## 7. O Fragment

**Scheme 13:**
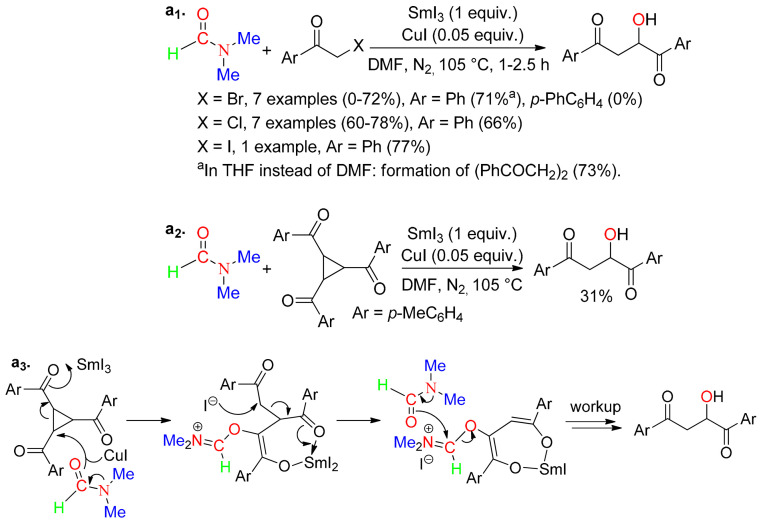
Hydroxylation reaction.

In DMF, the SmI_3_/CuI-promoted condensation of α-halo ketones resulted in an unexpected hydroxylation reaction (Scheme 13(a_1_)) [48]. Liu’s team demonstrated that the reaction arises via a tribenzoylcyclopropane, which affords the corresponding α-hydroxy-1,4-diketone (Scheme 13(a_2_)) through the participation of two DMF entities (Scheme 13(a_3_)).

**Scheme 14:**
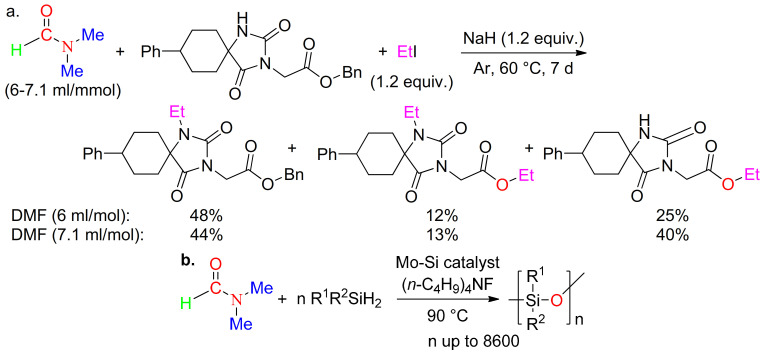
Oxygen insertion.

Zoidis and co-workers revealed a competing transesterification in the course of *N*-alkylation of *N*-(benzoxycarbonylmethyl)hydantoins with ethyl iodide and sodium hydride in DMF (Scheme 14a) [49]. According to the authors, the reaction occurs via attack of the DMF-zwitterionic mesomer **I_6_** on EtI, yielding the ammonium salt Me_2_N^⊕^=CHOEt which suffers from the addition of hydride leading to Me_2_NCH(OEt)H. Dissociation of the latter gives EtO^⊖^ which undergoes an S_N_2 reaction with the benzyl ester.

Catalysis with silyl-molybdenum complexes of the polymerization of dihydroorganosilanes in DMF led to the participation of the DMF-oxygen atom affording polysiloxanes (Scheme 14b) [50].

## 8. CO Fragment

A recent review summarizes carbonylations using diverse CO surrogates including DMF [51].

The use of DMF as the solvent has favored carbonylations with oxalic acid [52], Mo(CO)_6_ [53,54], or formylpyrrolidine [55] as the CO source, or carbon monoxide pressure [56].

DM was not the CO source of the amide functionality obtained from Pd-catalyzed hydrocarbonylation of alkenes in DM under CO pressure, only the NMe_2_ moiety was involved [3,57]. To the best of our knowledge, no new report concerns the carbonylation reaction using the CO fragment of DM.

## 9. CONMe_2_ Fragment

Examples leading to side compounds containing the CONMe_2_fragment are included in Section 15.

**Scheme 15:**
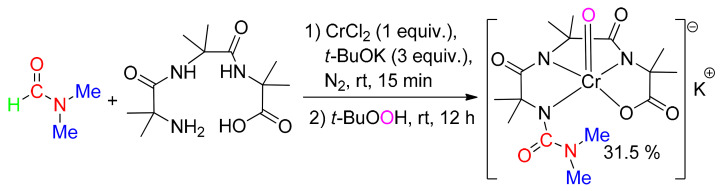
Formation of a 3,3-dimethylurea derivative.

In DMF, ligation to CrCl_2_ of the tripeptide formed from 2-amino-2-methylpropanoic acid followed by oxidation afforded the anionic urea Cr^V^ complex depicted in Scheme 15 [58].

**Scheme 16:**
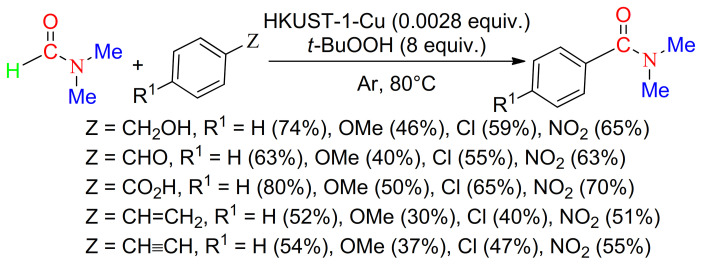
Oxidative cleavage of functionalized arenes.

The oxidation in DMF of benzyl alcohol, benzaldehyde, benzoic acid, styrene, phenyl acetylene, and corresponding *p*-substituted substrates using a mesoporous copper catalyst named HKUST-1-Cu led to cleavage of the Ar-function bond giving *N*,*N*-dimethylarylamides (Scheme 16) [59].

**Scheme 17:**
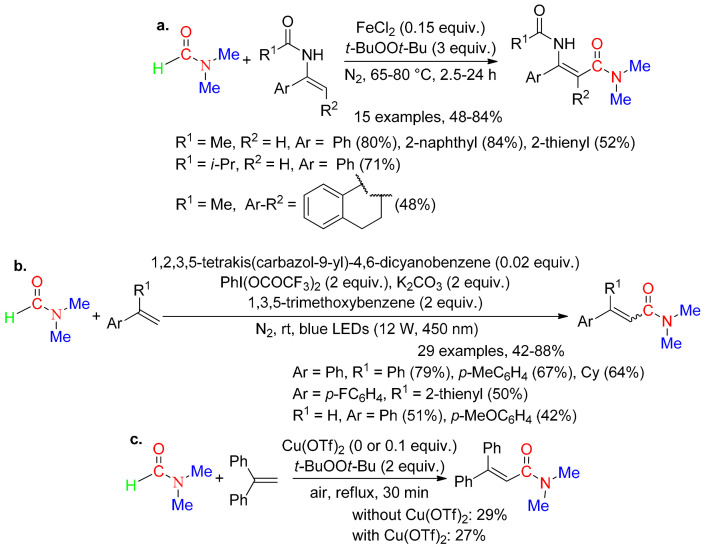
Carbamoylation of activated double bonds.

The carbamoylation of the double bond of enamides and styrenes with DMF arose under oxidative conditions mediated with Fe catalysis at 65–80 °C (Scheme 17a) [60] or visible light at room temperature (Scheme 17b) [61], respectively. Refluxing 1,1-diphenylethylene in DMF with di-*tert*-butyl peroxide led to a low yield of *N*,*N*-dimethyl-3,3-diphenylacrylamide even under Cu catalysis (Scheme 17c) [62].

**Scheme 18:**
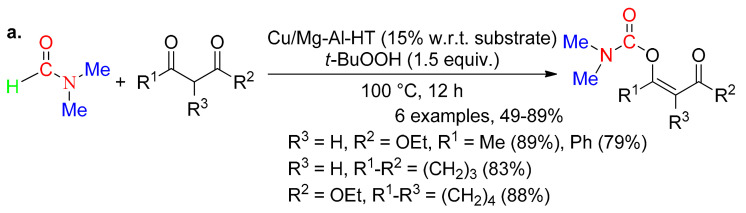

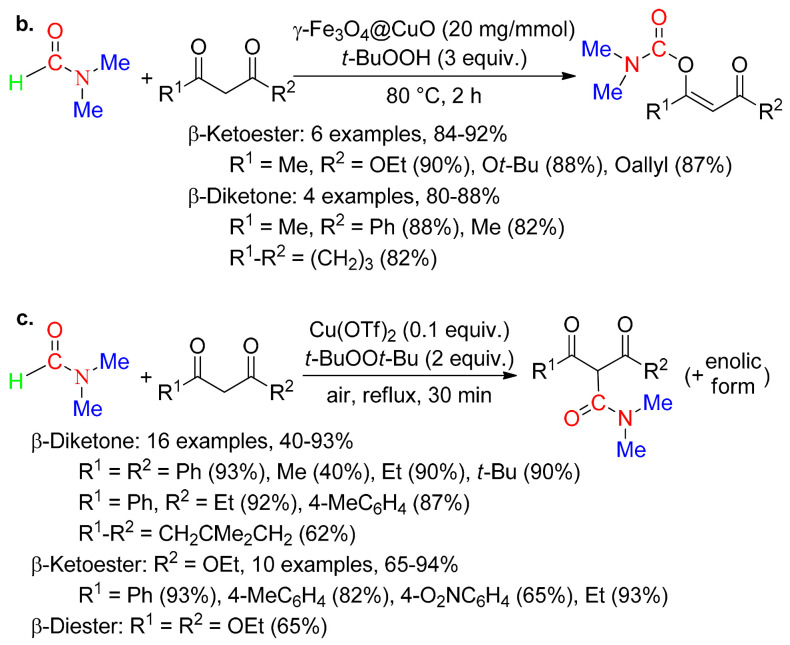
O- or C-carbamates from β-dicarbonyl compounds.

In the presence of peroxides, the reaction of β-dicarbonyl compounds with DMF under catalysis with either a copper supported Mg–Al hydrotalcite derived oxide (Scheme 18a) [63] or a maghemite–copper oxide nanocomposite (Scheme 18b) [64] afforded enol carbamates, while a soluble catalyst—Cu(OTf)_2_—gave, according to Zou’s team, 2-carbamoyl-1,3-dicarbonyl compounds (Scheme 18c) [62]. Given the NMR chemical shifts attributed to the latter, especially those of the putative 2-benzoyl-*N*,*N*-dimethyl-3-oxo-3-phenylpropanamide, the right structure is not obvious and remains an open question. Mail sent to J.-P. Zou remained without an answer.

**Scheme 19:**
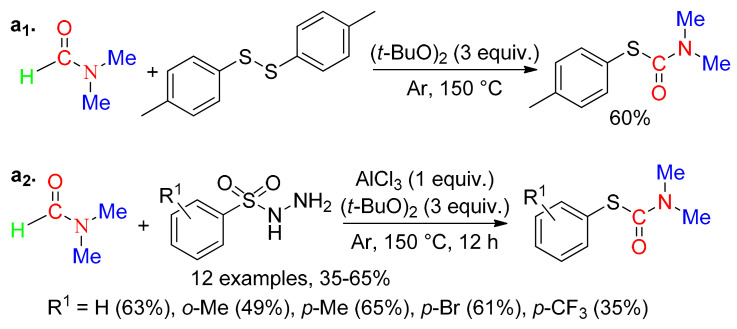

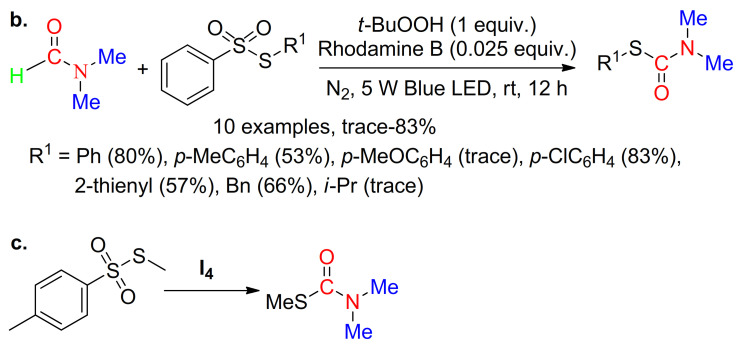
Thiolation of DMF.

Mao, Zhang, and co-workers reported the thiolation of the C–H bond of DMF using di-*tert*-butyl peroxide and either 1,2-di-*p*-tolyldisulfane (Scheme 19(a_1_)) or arylsulfonyl hydrazides and aluminum chloride (Scheme 19(a_2_)) [65], while Bi, Feng, Geng, and co-workers recently used *tert*-butyl hydroperoxide and *S*-aryl arenethiosulfonates under visible-light irradiation (Scheme 19b) [66]. This last procedure is, however, ineffective for DMF thiolation with 1,2-di-*p*-phenyldisulfane [66]. According to the authors [65,66], these reactions involve the radical **I_4_**. Jia’s team, in the course of the study of functionalization of styrenes with thiosulfonates and arylboronic acids in acetone/DMF, also proposed that *S*-methyl dimethylcarbamothioate isolated as a by-product was formed from the reaction of *S*-methyl 4-methylbenzenesulfonothioate with **I_4_** (Scheme 19c) [67].

**Scheme 20:**
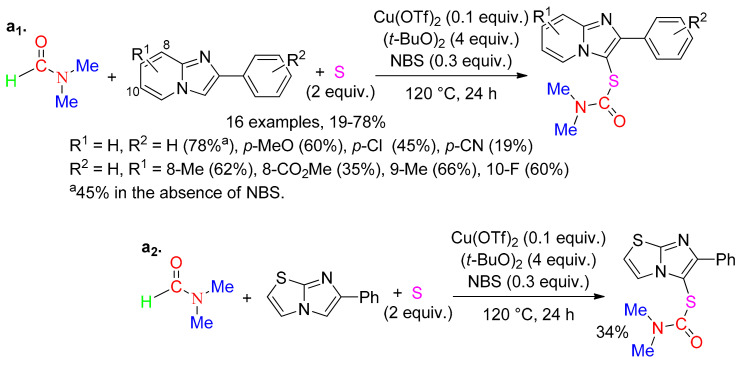
Thiocarbamation of heterocycles.

Thiocarbamation of 2-arylimidazo[1,2-*a*]pyridines with elemental sulfur and DMF has been carried out with di-*tert*-butyl peroxide and Cu catalysis at 120 °C [68]. The reaction was promoted with *N*-bromosuccinimide (Scheme 20(a_1_)) and also occurred using 6-phenylimidazo[2,1-*b*]thiazole as the substrate (Scheme 20(a_2_)). The Cui/Tang team proposed a radical mechanism with **I_4_** reacting with sulfur leading to Me_2_NCOS^∙^, and possibly also NBS giving Me_2_NCOSBr. Both species could react with the substrate to deliver the isolated product.

All the above processes of this section involve the participation of radical **I_4_** formed by catalyst/peroxide-mediated homolytic cleavage of DMF.

**Scheme 21:**
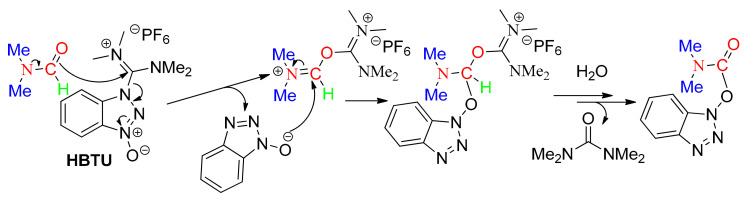
HBTU degradation.

Hexafluorophosphate benzotriazole tetramethyl uronium (HBTU) is a peptide coupling reagent frequently used [69,70,71]. An impurity with an abundance of 0.09% was detected by Badalassi and co-workers in the Peptide Q API solution obtained from the HBTU-mediated Peptide Q cyclization in DMF [72]. This impurity was identified as being 1*H*-benzo[*d*][1–3]triazol-1-yl dimethylcarbamate formed from the addition of DMF to HBTU as depicted in Scheme 21.

## 10. H Fragment

**Scheme 22:**
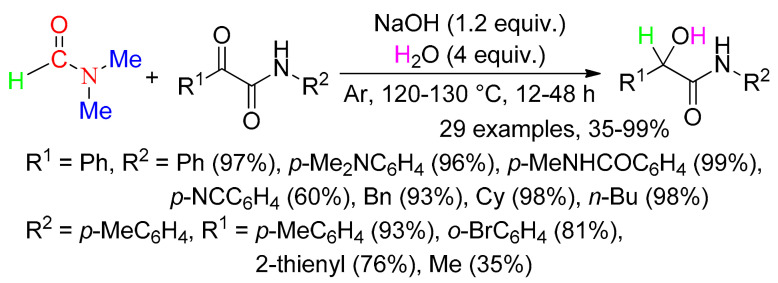
Hydride transfer.

The chemoselective reduction of α-ketoamides arose from treatment with sodium hydroxide and water in DMF (Scheme 22) [73]. Deuterium labeling experiments led Wu and co-workers to assume hydride delivery from HCOONa produced from the hydrolysis of DMF with hydrated NaOH.

**Scheme 23:**
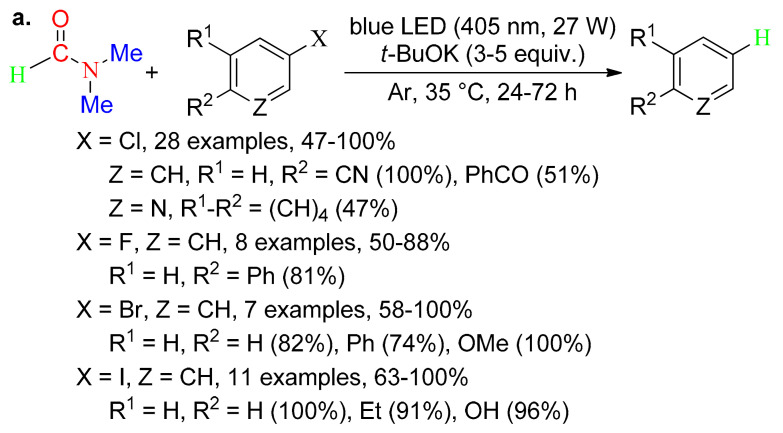

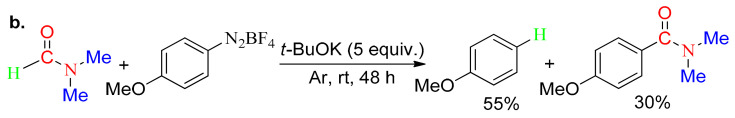
Radical reactions.

The association of *t*-BuOK, DMF, and visible-light irradiation mediated the dehalogenation of (hetero)aryl fluorides, chlorides, bromides, and iodides (Scheme 23a) [74], while the reductive cleavage of 4-methoxybenzenediazonium tetrafluoroborate occurred under light-free conditions (Scheme 23b) [75]. According to Qu/Kang’s and Taillefer’s teams [74,75], these reactions implicate the carbamoyl anion **I_7_** and, via electron transfer, the carbamoyl radical **I_4_**. Such a reactivity of DMF under *t*-BuOK conditions has been exploited for transition-metal-free Matsuda–Heck type reactions [75].

## 11. RC Fragment

**Scheme 24:**
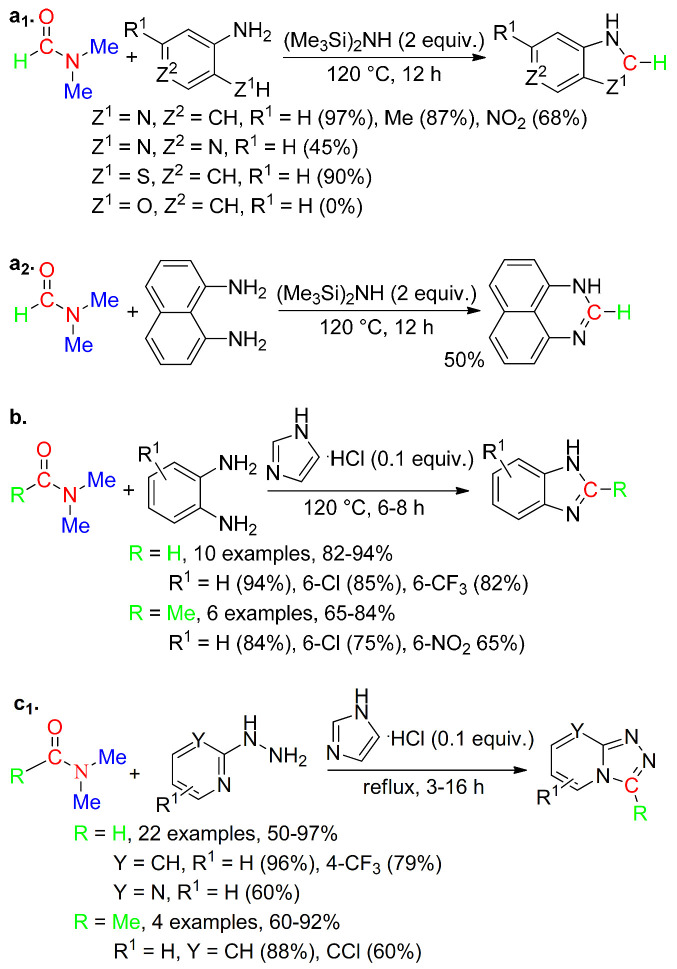

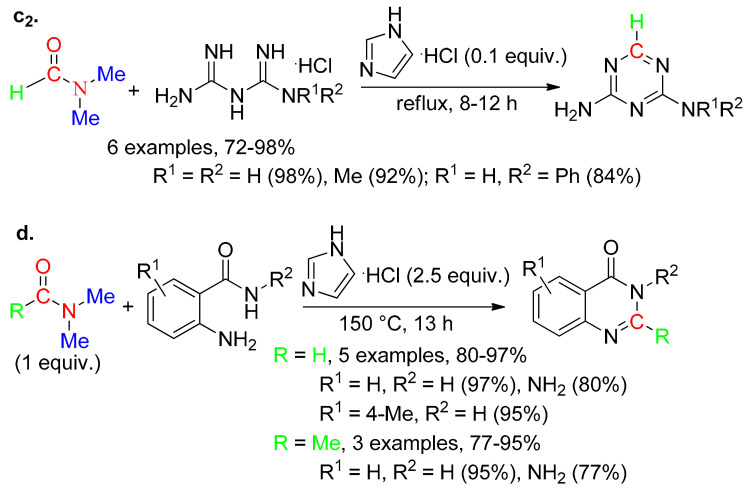
Heterocyclization involving CR.

Heterocycles containing the CR fragment have been synthetized from a variety of amino substrates (Scheme 24) [76,77,78,79]. Under the experimental conditions of Scheme 24a, activation of the carbonyl group of DMF by HMDS favors the nucleophilic addition of the arylamine leading to an intermediate that undergoes internal addition of the other nucleophilic moiety of the substrate. In the presence of imidazolium chloride (Scheme 24b–d), the addition of imidazole to HCl-activated carbonyl of DM leads to C_3_H_3_N_2_CO**R** which undergoes nucleophilic addition with the substrate.

**Scheme 25:**
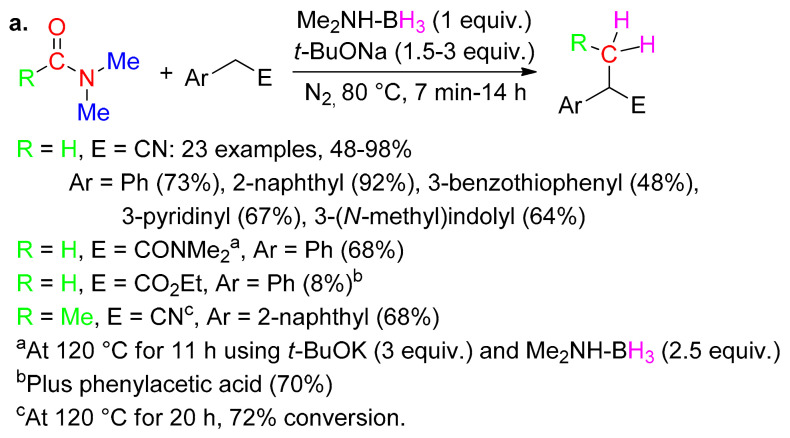

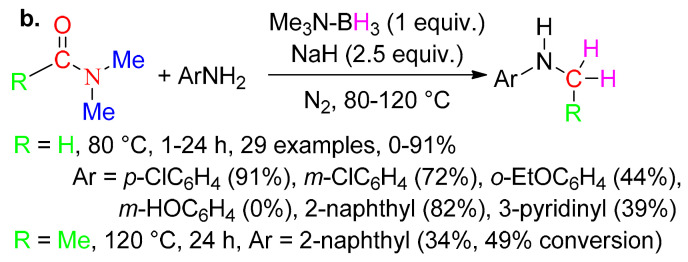
*C*- and *N*-methyl and ethylations.

**Scheme 26:**
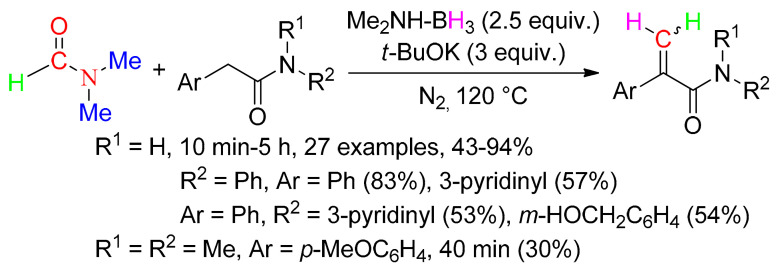
Direct Csp^3^ methylenation.

Using both *t*-BuONa and amine-borane in DM, Y.-F., Wang, and co-workers carried out the *C*-methylation or *C*-ethylation of *N*,*N*-dimethyl-2-phenylacetamide and various arylacetonitriles in fair to high yields while the reaction of ethyl 2-phenylacetate was much less efficient (Scheme 25a) [80]. One year later, in collaboration with the team of Z. Wang, they disclosed the methylenation of 2-arylacetamides under similar conditions (Scheme 26) [81]. Another year later, they reported the *N*-monomethylation and *N*-monoethylation of primary anilines with the NaH/Me_2_NH-BH_3_/DM association (Scheme 25b) [82]. Interestingly, the use of DMF or *d_7_*-DMF with Me_2_NH-BH_3_ and Me_2_NH-BD_3_ allowed the controllable installation of *N*-CH_2_D, *N*-CHD_2_, and *N*-CD_3_ units [82]. The proposed *N*-methylation mechanism of anilines includes deprotonation of ArNH_2_ mediating addition to DM leading to anionic species ArNHCRO^⊖^NMe_2_ which evolves toward ArN=CRNMe_2_. The ensuing reduction with Me_2_NH-BH_3_ provides ArNHCHRNMe_2_ [82]. The base-promoted elimination of HNMe_2_ gives ArN=CHR which undergoes reduction leading to ArNH(CH_2_R).

**Scheme 27:**
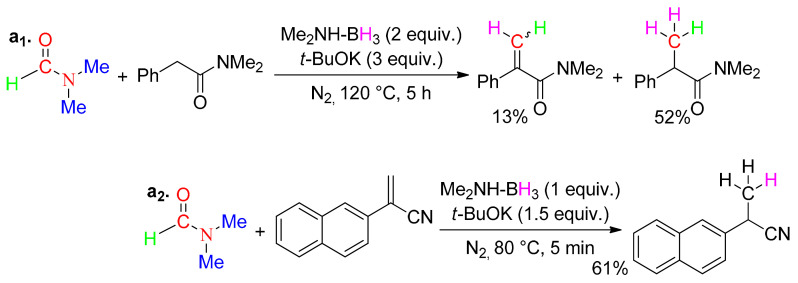
Methylation versus methylenation and hydrogen transfer.

The difference between the results—methylation versus methylenation—of *N*,*N*-dimethyl-2-arylacetamides (Scheme 25a versus Scheme 26) is surprising but the authors did not cite the first report and, consequently, did not make comments. *N*,*N*-Dimethyl-2-phenylacetamide (Scheme 25a) [80] and 2-(4-methoxyphenyl)-*N*,*N*-dimethylacetamide (Scheme 26) [81] were treated at 120 °C with same amounts of *t*-BuOK and amine-borane. Y.-F. Wang’s team assumed that the methylation product implicates the reduction of the methylenation product [80]. According to the two reports [80,81], deprotonation of the substrate (noted ArCH_2_E) mediates addition to DMF leading to ArECHCH(OH)NMe_2_ which evolves toward the imminium ArECHCH=N^⊕^Me_2_. Subsequent reduction with Me_2_NH-BH_3_ into ArECHCHH(NMe_2_) is followed by base-mediated elimination of HNMe_2_ leading to ArEC=CHH and then the methylation product. The nature of the final product could depend on the reaction time. Indeed, the methylenation product was isolated after 40 min while the methylation reaction went on for 11 h. Moreover, decreasing the reaction time to 5 h afforded a mixture of the two products (Scheme 27(a_1_)) [80]. The hypothesis of the effect of the reaction time, however, disagrees with the reduction of acrylonitrile in 5 min under the *t*-BuOK/Me_2_NH-BH_3_/HCONMe_2_ conditions (Scheme 27(a_2_)) [80]. In conclusion, the interpretation of the results depicted in Scheme 25a and Scheme 26 remains an open question.

**Scheme 28:**
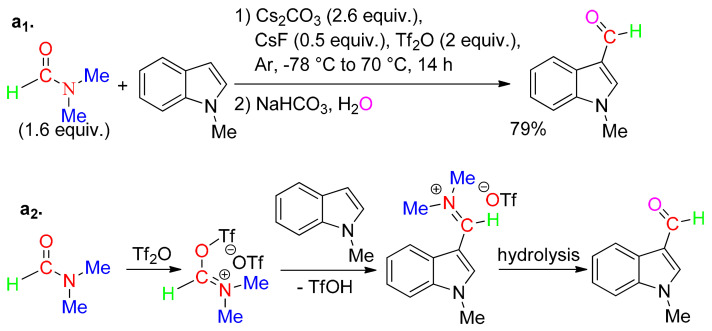
Tf_2_O-mediated formylation of *N*-methylindole.

Liang’s team revealed the triflic anhydride-mediated formylation of *N*-methylindole depicted in Scheme 28(a_1_) [83]. The reaction proceeds through nucleophilic addition of the substrate to enolium triflate of DMF giving the corresponding iminium (Scheme 28(a_2_)) [83,84]. Subsequent hydrolysis delivers the product.

## 12. HCNMe_2_ Fragment

**Scheme 29:**
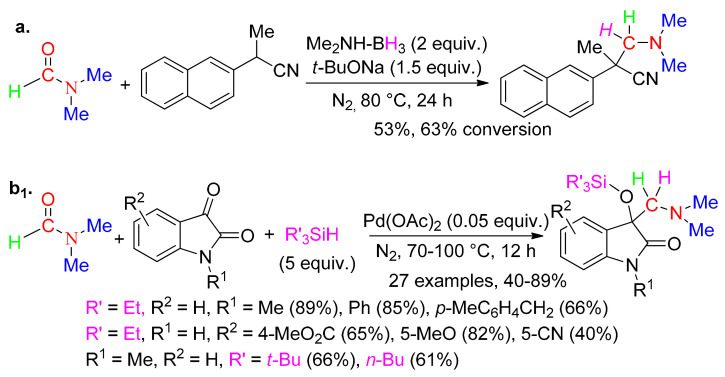

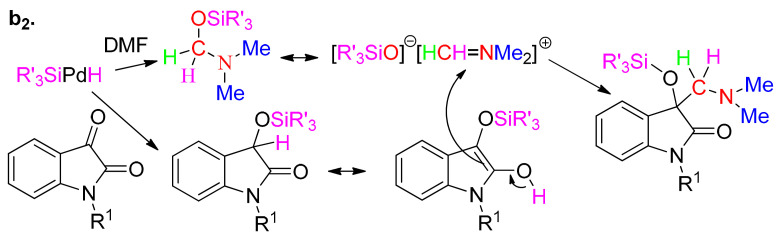
Substitution of a tertiary Csp3–H or addition to a carbonyl group.

Mechanistic investigations of the reaction depicted in Scheme 25a led Y.-F. Wang’s team to observe a slow reaction of the methylation product with the amine-borane/DMF system (Scheme 29a) [80]. The iminium intermediate ArCMe(CN)CH=N^⊕^Me_2_, produced by deprotonation of the substrate and addition to DMF as documented above, is reduced with amine-borane.

The 3-carbonyl group of isatins underwent both hydrosilylation and amino-methylation using hydrosilanes and Pd catalysis in DMF (Scheme 29(b_1_)) [85]. According to Wu’s team, Pd^0^ produced from the silane-mediated reduction of Pd(OAc)_2_, inserts into the Si–H bond of the silane giving R’_3_SiPdH, which undergoes two different hydrosilylations (Scheme 29(b_2_)). That of DMF affords (R’_3_SiO)HCHNMe_2_ which transforms into [(R’_3_SiO)]^⊖^[HCH=NMe_2_]^⊕^, while that of the C3 carbonyl of isatin provides *O*-silylated indolin-2-one which tautomerizes into the corresponding silyl enol ether. The addition of the latter to the iminium species gives the product.

## 13. HC-O Fragment

**Scheme 30:**
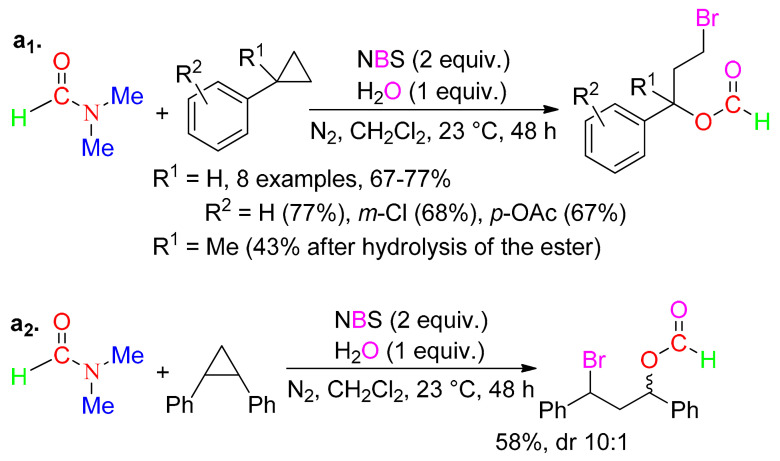
1,3-difunctionalization of aryl cyclopropanes.

1,3-Bromoesters have been isolated from the reaction of aryl cyclopropanes with NBS, DMF, and H_2_O (Scheme 30(a_1_)), which involves bromination of the three-membered ring leading to carbocation ArR^1^C^⊕^CH_2_CH_2_Br [86]. The subsequent attack of the oxygen of DMF via S_N_1 or S_N_2 mechanism affords ArR^1^C(OCH=N^⊕^Me_2_)(CH_2_CH_2_Br) which undergoes hydrolysis delivering the bromoester. The reaction of 1,2-diphenylcyclopropane under the same experimental conditions arose with good diastereoselectivity (Scheme 30(a_2_)).

## 14. RC=O Fragment

**Scheme 31:**
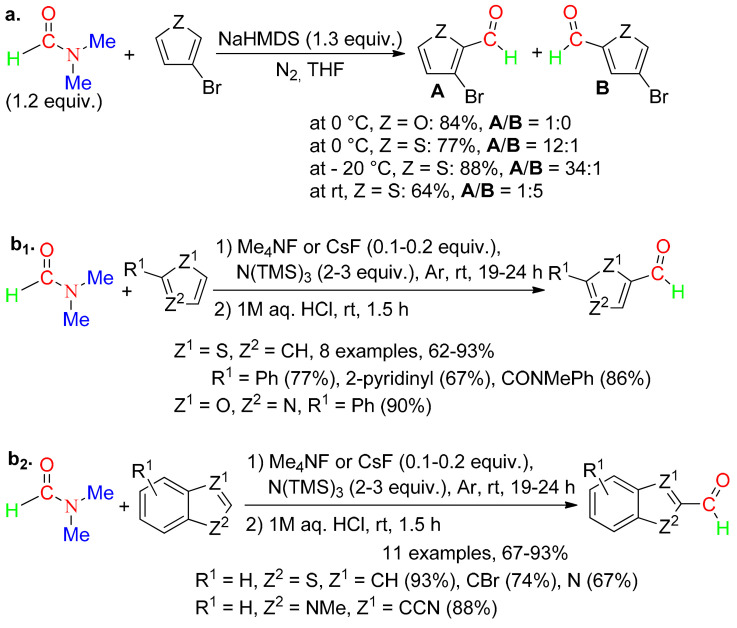
Formylation of 5-membered heteroarenes.

The regioselective 2-formylation of 3-bromobenzofuran and 3-bromobenzothiophene was achieved with NaHMDS and DMF at low temperature in THF, while the C5 position was favored at room temperature (Scheme 31a) [87]. Formylation of a variety of five-membered heteroarenes succeeded at room temperature with in situ generated amide base (Scheme 31b) [88]. The latter reactions proceed via proton abstraction and nucleophilic addition of the resulting carbanion to DMF.

**Scheme 32:**
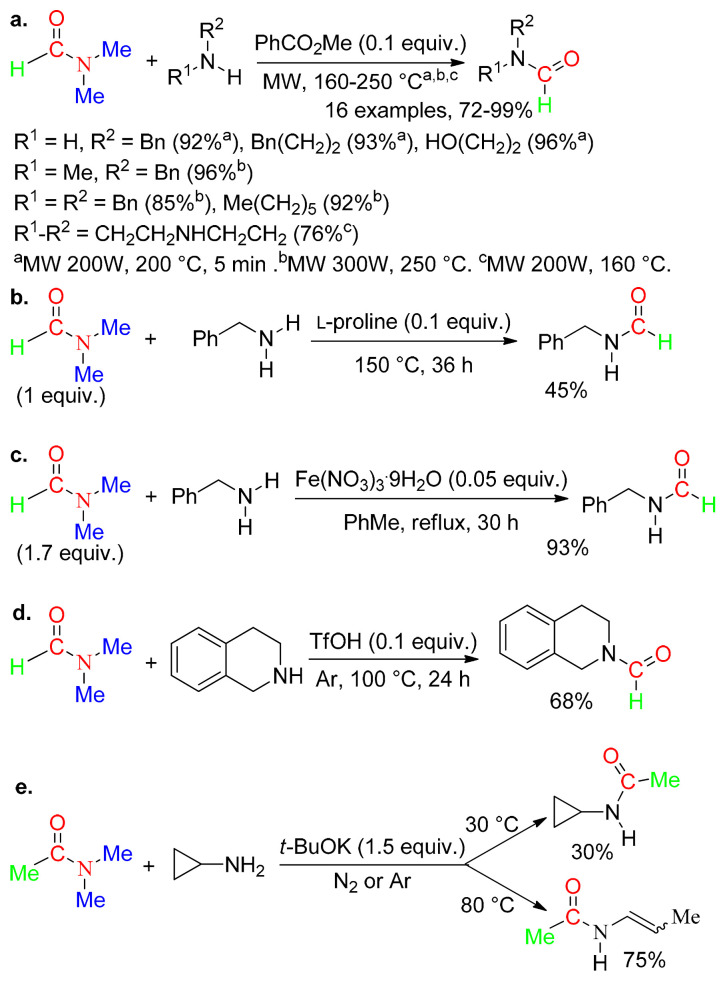
Amidation of aliphatic amines.

**Scheme 33:**
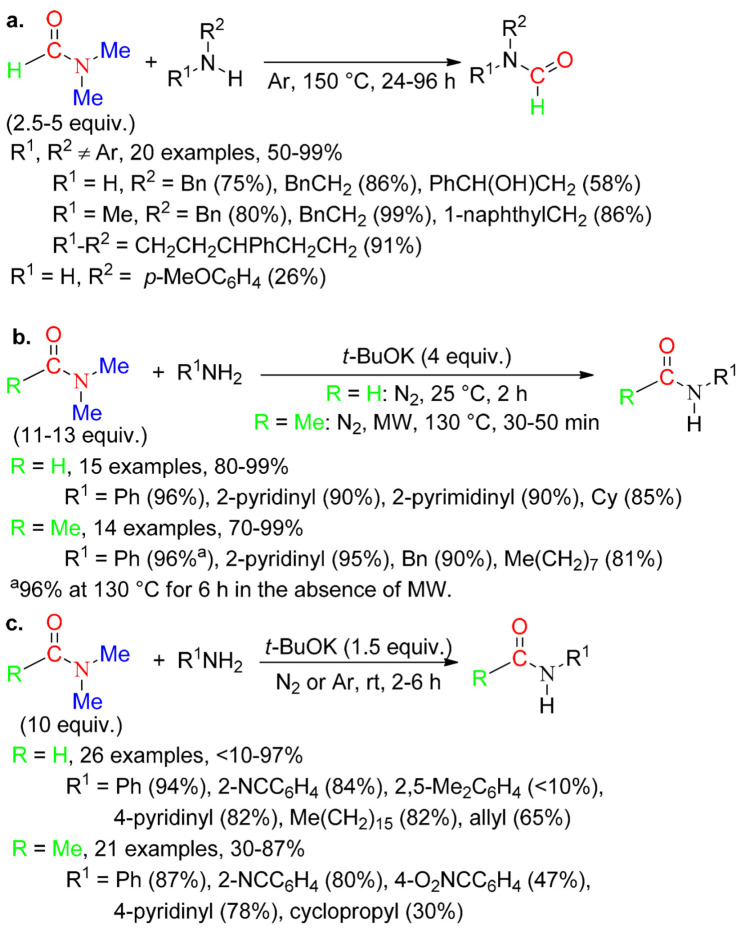

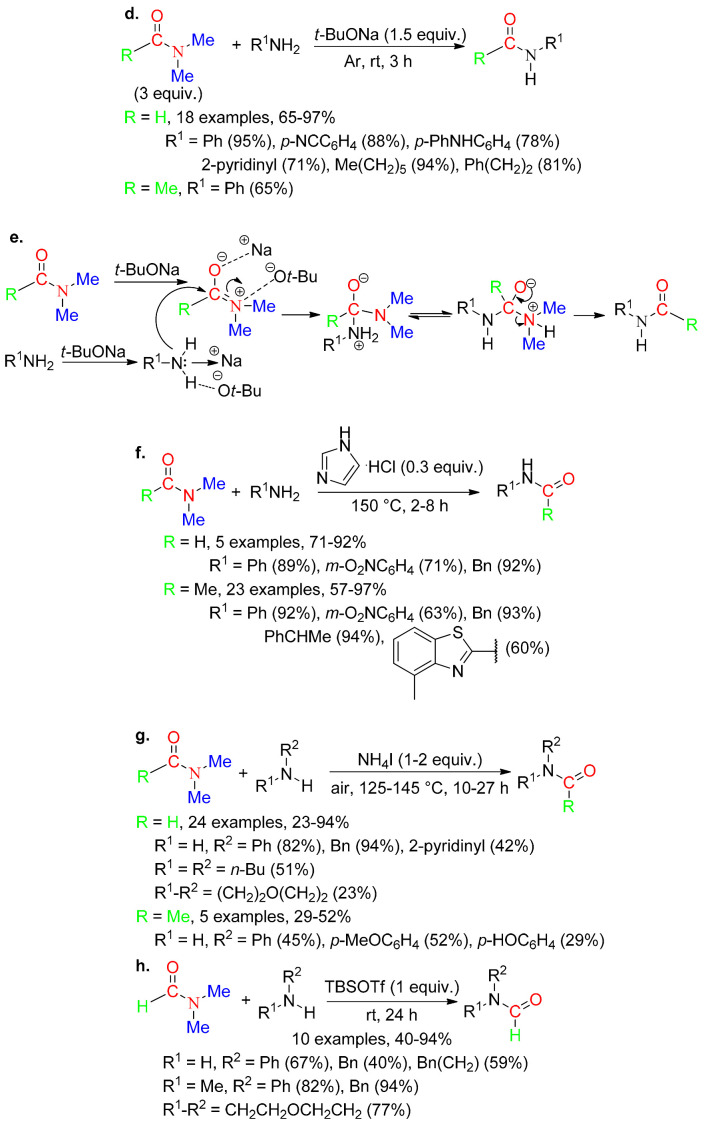

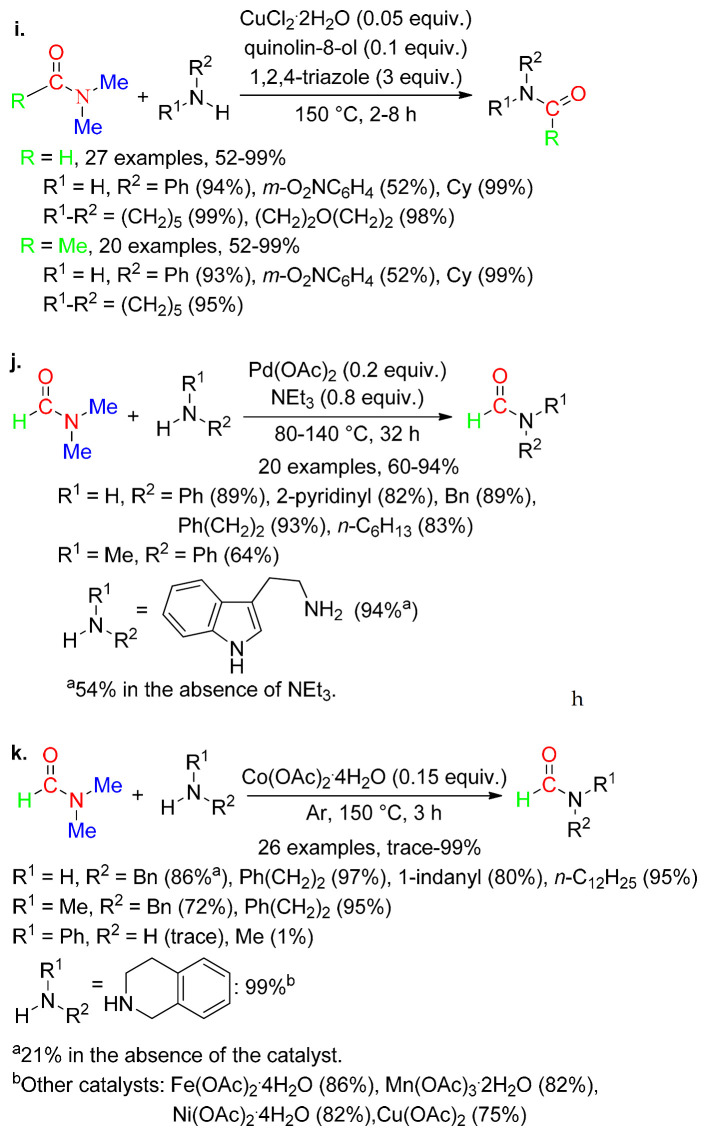
Amidation of aliphatic amines and arylamines.

The transamidation of amines has been carried out under a variety of conditions [89]. A rather surprising procedure using methyl benzoate under microwave irradiation in DMF achieved the formylation of aliphatic primary and secondary amines (Scheme 32a) [90]. Jeon and Yang speculated a transition state implicating amine, methyl benzoate, and DMF. L-Proline at 150 °C [91] and Fe^III^ salts in refluxing toluene [92] were used to catalyze the formylation of benzylamine with DMF (Scheme 32b,c). The reaction of L-proline with DMF would precede the nucleophilic addition of the amine, while Fe^III^ would form a DMF complex that reacts with the amine. Triflic acid catalyzed the formylation of tetrahydroisoquinoline with DMF (Scheme 32d) [93]. In fact, formylations with DMF arose at 150 °C even in the absence of an additive, in fair to high yields from various aliphatic amines and a low yield from *p*-methoxyaniline (Scheme 33a) [94].

Potassium and sodium *tert*-butoxides mediated the formylation and acetamidation of primary aliphatic and arylamines with DM (Scheme 33b–d) [95,96,97]. The teams of Dash [96] and Cheng/Chen [97] performed the reactions with DMF and DMAc at room temperature (Scheme 33c,d), while, in contrast to DMF, the Li/Yu team carried out the reactions with DMAc at 130 °C and under microwave irradiation to reduce the reaction time (Scheme 33b) [95]. The *t*-BuOK-mediated reaction of cyclopropylamine provided the transamidation product in a poor yield at room temperature and *N*-(prop-1-en-1-yl)acetamide in fair yield at 80 °C (Scheme 32e) [96]. Effective transamidations of primary amines with DM were reported under catalysis with imidazolium chloride at 150 °C (Scheme 33f) [98] or using 1–2 equiv. of ammonium iodide at 125–145 °C (Scheme 33g) [99].

Basic conditions implicate the deprotonation of the amine and the tetrahedral ionic species (R^1^HN)CRO^⊖^(NMe_2_) which converts into the product. According to the Li/Yu team, the mechanism depends on the nature of the base: no radical character of the *t*-BuONa-based reaction while two pathways were plausible with *t*-BuOK [95]. With *t*-BuOK, (R^1^HN)CRO^⊖^(NMe_2_) would be obtained either from the reaction between R^1^HN^⊖^ and a **I_6_***/t*-BuOK complex or via the equilibrium R^1^HN^⊖^ 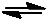 R^1^HN**^∙^** + e^⊖^, allowing a single electron transfer to form the radical anion **I_8_** (RC**^∙^**O^⊖^(NMe_2_)). Coupling of the latter with R^1^HN**^∙^** leads to (R^1^HN)CRO^⊖^(NMe_2_). Such a radical pathway agrees with subsequent EPR and ESI-MS studies performed by Dash’s team [96]. For the *t*-BuONa-based reaction, Cheng/Chen team proposed a mechanism without single-electron transfer (Scheme 33e). Both DM and amine are separately activated via coordinative interactions with *t*-BuONa. The reaction between the two entities leads to a tetrahedral intermediate which undergoes proton transfer. Subsequent elimination of HNMe_2_ delivers the desired amide [97]. The difference between the *t*-BuOK and *t*-BuONa reaction pathways was assigned to the stronger basicity of the former and its good single-electron transfer properties [97].

The imidazolium chloride-based reaction (Scheme 33f) would involve the protonation of DM, promoting the nucleophilic addition of imidazole leading to C_3_H_3_N_2_COR. Subsequent amine addition gives C_3_H_3_N_2_C(OH)R(NHR^1^) which evolves toward the product via elimination of imidazole.

**Scheme 34:**
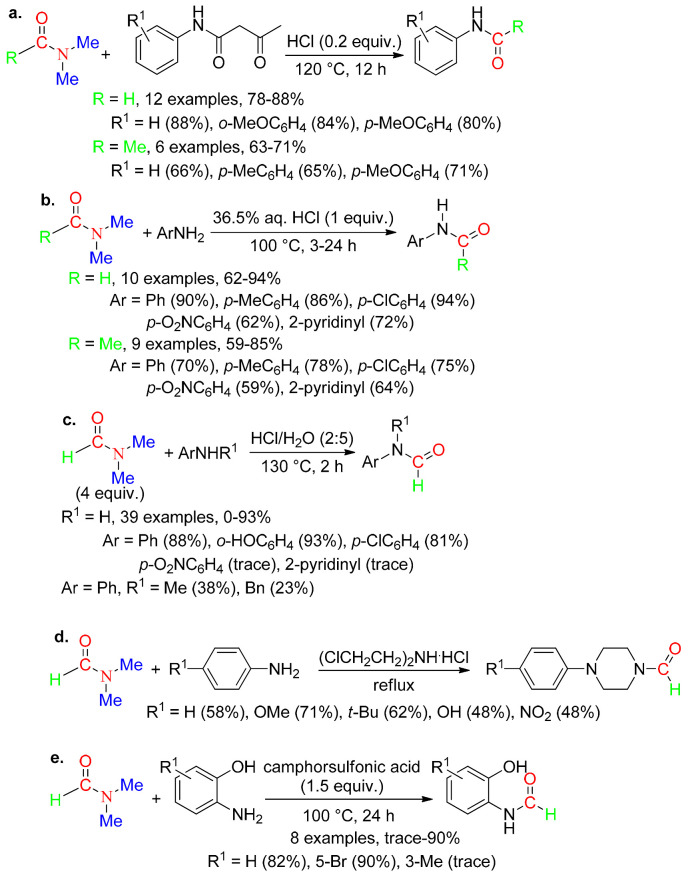

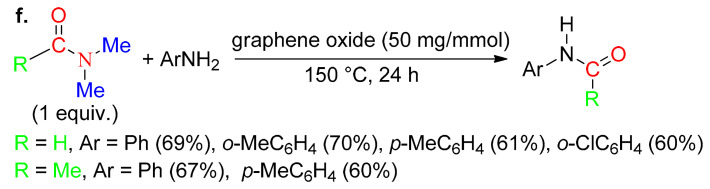
Amidation of arylamines.

Various acidic conditions in DM, in particular HCl catalysis at 120 °C, achieved the formation of *N*-phenylamides from β-ketobutylanilides (Scheme 34a) [100]. According to Chen’s team, the substrate decomposes into the corresponding anilide which undergoes reaction with protonated DMF. In fact, the same laboratory subsequently reported the amidation of primary arylamines using a stoichiometric amount of aqueous HCl in DM at 100 °C (Scheme 34b) [101]. Recently, Karpoormath’s team used a similar procedure for amidation with DMF of primary and secondary amines (Scheme 34c) [102]. Martínez-Pascual and co-workers, who performed the formylation of anilines and secondary aliphatic amines using the beforehand prepared DMF^.^HCl complex, reported a domino reaction leading to 4-arylpiperazine-1-carbaldehydes from anilines, bis(2-chloroethyl)amine hydrochloride, and DMF (Scheme 34d) [103]. Camphor sulfonic acid was the optimum carboxylic acid for *N*-formylation of 2-aminophenols at 100 °C (Scheme 34e) [104]. In contrast, Lewis acids such as *tert*-butyldimethylsilyl triflate promoted the room temperature formylation of primary or secondary aliphatic amines and anilines (Scheme 33h) [105]. Heating was required for the amidation of arylamines mediated with graphene oxide under neat conditions (Scheme 34f) [106].

*N*-Amidation of aryl and aliphatic amines arose in high yields at 80–150 °C under CuCl_2_ (Scheme 33i) [107] or PdCl_2_ catalysis (Scheme 33j) [108] while Co(OAc)_2_ as catalyst was efficient only from aliphatic amines (Scheme 33k) [109]. The Cu-catalyzed reaction was carried out in the presence of 1,2,4-triazole. Jagtap’s team assumed that this additive undergoes addition to Cu^II^-coordinated DM, resulting in the formation of HNMe_2_ and Cu^II^-coordinated (1,2,4-triazol-1-yl)COR. Nucleophilic addition of R^1^R^2^NH to the latter followed by elimination of 1,2,4-triazole and Cu^II^ affords the product [107]. Such a pathway contrasts with the proposal of Gong and co-workers who assumed the direct amine addition to the DMF/Co^II^ complex [109] as above alleged under Fe^III^ catalysis [92]. No mechanism was indicated by L. Zhang’s team for the Pd-catalyzed reaction [108]; the yield decreased in the absence of NEt_3_ (Scheme 33j), leading us to suspect a pathway similar to that mediated by Cu^II^.

**Scheme 35:**
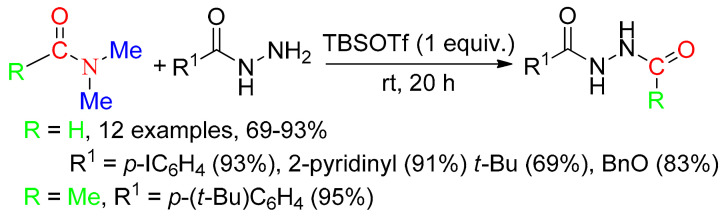
Formylation and acetylation of hydrazides.

Formylation and acetylation of hydrazides with *tert*-butyldimethylsilyl triflate and DM effectively occurred at room temperature (Scheme 35) [105].

**Scheme 36:**
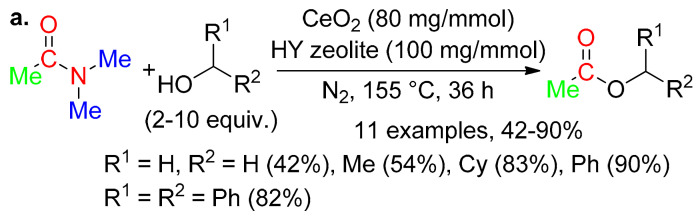

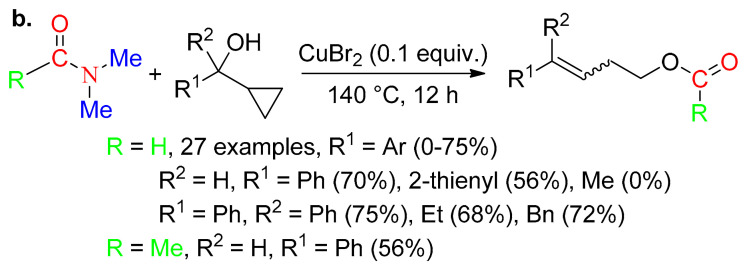
CeO_2_-promoted alcoholysis of DMAc and CuBr_2_-catalyzed ring opening/esterification of cyclopropyl carbinols.

Siddiki, Shimizu, and co-workers disclosed the esterification of primary and secondary alcohols with DMAc using CeO_2_ at 155 °C in the presence of HY zeolite (SiO_2_/Al_2_O_3_) (Scheme 36a) [110]. HY zeolite, enclosed in a paper filter placed at the upper portion of the reaction vessel, traps the dimethylamine formed from the CeO_2_-promoted cleavage of the C–N bond of DMAc, which concomitantly affords a CeOCO**Me** species. Nucleophilic addition to the latter of the alcoholate formed from CeO_2_-mediated deprotonation of the alcohol provides the ester.

Treatment at 140 °C in DM of cyclopropyl carbinols with catalytic amounts of CuBr_2_ provided alk-3-en-1-yl formates or acetates (Scheme 36b) via a copper alkoxide complex which evolves toward a homoallylic copper alcoholate [111]. Nucleophilic attack of the latter on DM results in the formation of the ester.

The formyl moiety of DMF would be involved in the formation of benzyl formate identified as a side reaction of the oxidation of benzyl bromide with a Zr-photocatalyst in DMF under air atmosphere [112].

**Scheme 37:**
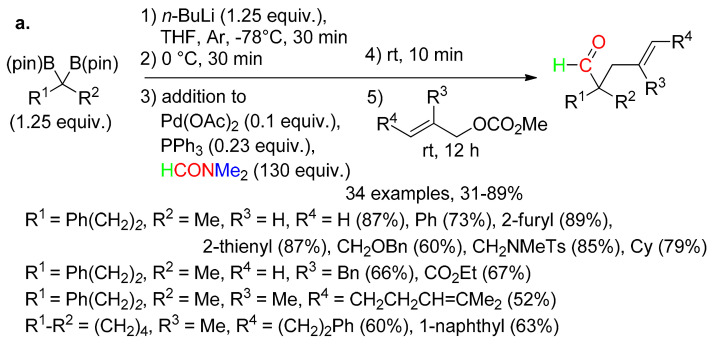

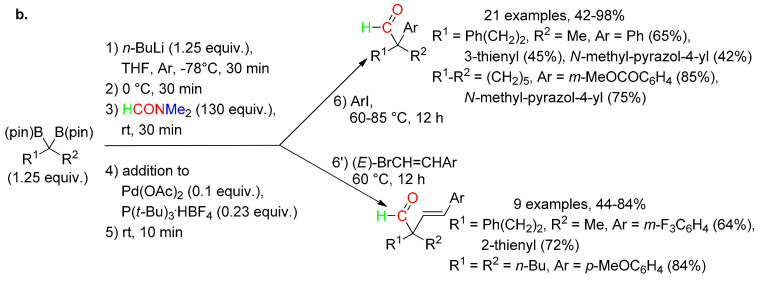
One-pot reactions.

Quaternary carbons have been synthetized from *gem*-bis(boronates), DMF and allyl methyl carbonates [113], (hetero)aryl iodides, or alkenyl bromides [114] using the procedures disclosed in Scheme 37. According to Xu and co-workers, the lithium salt obtained from treatment of R^1^R^2^C[B(pin))]_2_ with *n*-BuLi, reacts with DMF to afford R^1^R^2^C=CH[OB(pin)]. Transmetallation with R’PdX (R’ = substituted allyl, Ar, CH=CHAr) leads to R^1^R^2^C=CH(OPdR’) which is in equilibrium with the tetrahedral intermediate R^1^R^2^C(PdR’)(CH=O). Then, reductive elimination of Pd^0^ liberates R^1^R^2^CR’(CH=O).

## 15. RC=ON(CH_2_)Me Fragment

**Scheme 38:**
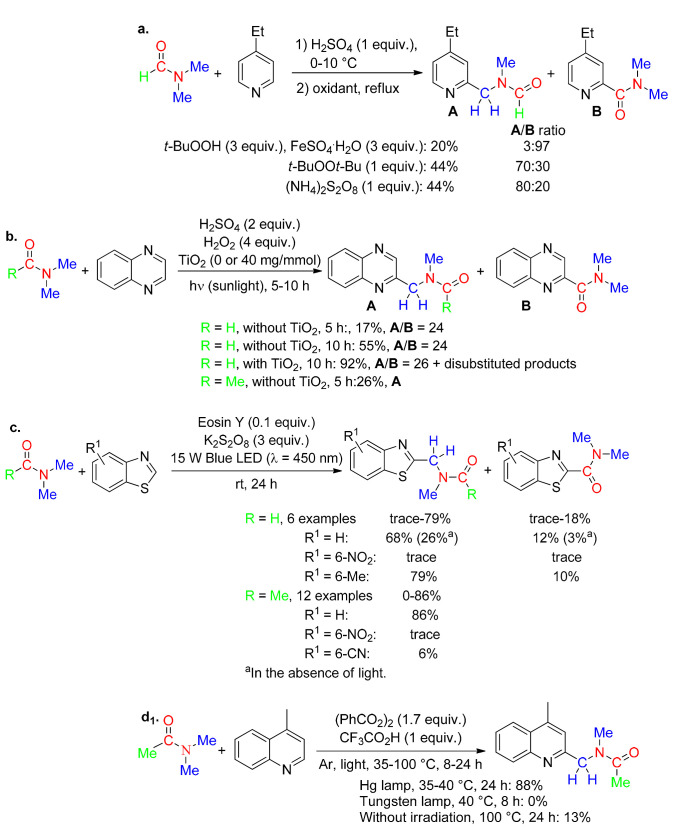

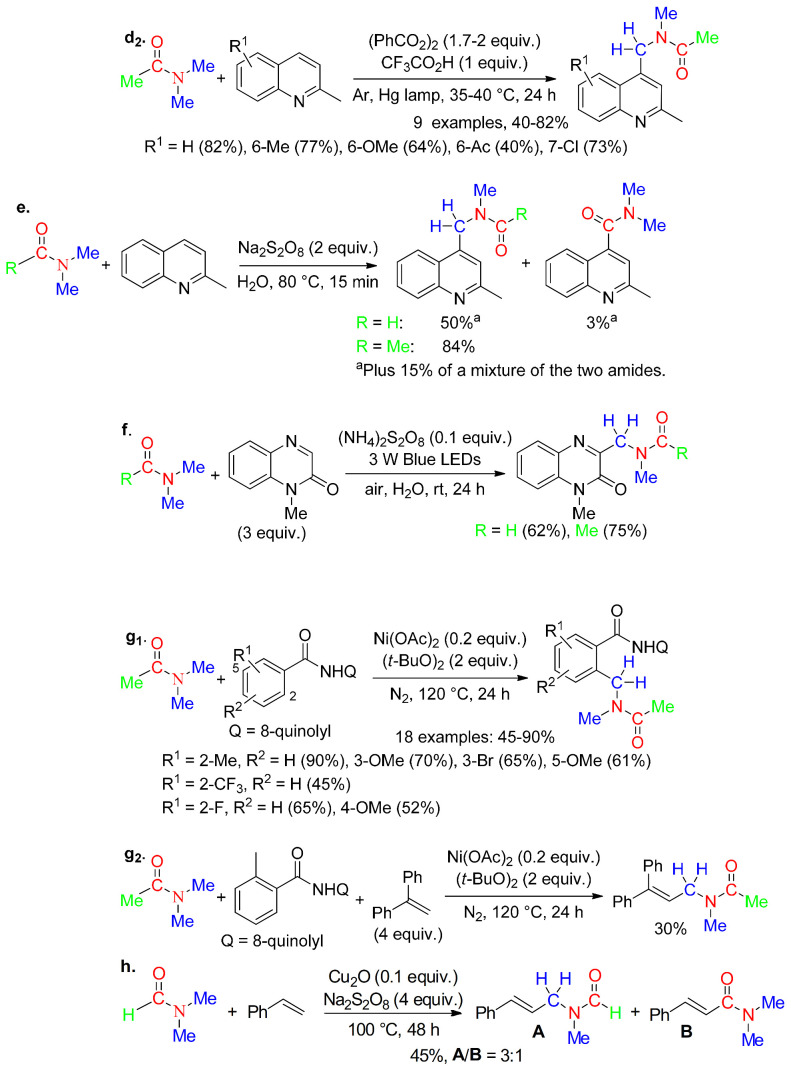

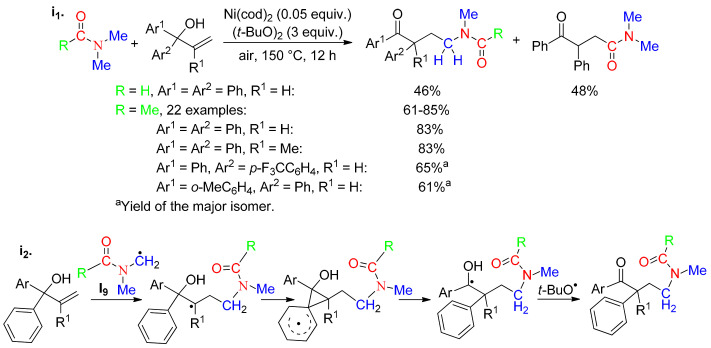
Carbamoylation versus amidation.

In 1976, Minisci’s team disclosed the reaction of heteroarenes with DMF in the presence of sulfuric acid and oxidants [115]. Thus, 4-ethyl pyridine provided a mixture of 4-ethyl-*N*,*N*-dimethylpicolinamide and *N*-((4-ethylpyridin-2-yl)methyl)-*N*-methylformamide in yields and ratios depending on the oxidant (Scheme 38a). This seminal report was followed by intensive studies on the radical fragmentation of DM by Minisci and co-workers [116,117,118]. Then, the promotion of such reactions under sunlight, especially in the presence of TiO_2_, was disclosed by Caronna and co-workers (Scheme 38b) [119]. Subsequently, Weng’s team reported that the amidoalkylaion method reported by the Huang/Zhu team [120] was improved using a photocatalyst and visible light (Scheme 38c) [121]. Togo’s team previously highlighted the decisive effect of UV light on the amidoalkylation of 4-methylquinoline using DMAc, benzoyl peroxide, and trifluoroacetic acid (Scheme 38(d_1_)) [122]. Various quinolines (Scheme 38(d_2_)), isoquinolines, and phenanthridines were amidoalkylated under such conditions [122]. Then, Gambarotti and Truscello reported oxidative conditions with sodium persulfate in water leading to short reaction times in the absence of acids (Scheme 38e) [123]. Water as the solvent was also subsequently used by J. Li’s team but with catalytic ammonium persulfate under oxygen atmosphere and light assistance for the C-3 functionalization of 1-methylquinoxalin-2(1*H*)-one by DMF or DMAc (Scheme 38f) [124]. The teams of Han and Y. Zhang used the chelating properties of the 8-aminoquinolyl group for the regioselective Ni^II^-catalyzed coupling of *N*-(quinolin-8-yl)benzamides with DMAc, the use of di-*tert*-butyl peroxide as oxidant leading to selective reaction of a C(sp^3^)−H bond adjacent to nitrogen of DMAc (Scheme 38(g_1_)) [125]. The Ni^II^-catalyzed selective carbamoylation of 1,1-diphenylethene with DMAc under peroxide conditions in the presence of 2-methyl-*N*-(quinolin-8-yl)benzamide (Scheme 38(g_2_)) revealed the radical character of the process [125]. Under Cu_2_O catalysis, oxidation of styrene with Na_2_S_2_O_8_ in DMF provided a 3:1 mixture of *N*-cinnamyl-*N*-methylformamide and *N*,*N*-dimethylcinnamamide (Scheme 38h) [126]. The Ni(cod)_2_/*t*-BuOOH association in DMAc mediated the selective carbamoylation of α,α-diaryl allylic alcohols while amidation was a competitive pathway in DMF (Scheme 38(i_1_)) [127]. Both pathways involve a radical 1,2-aryl migration succeeding to the reaction of the substrate with either **I_9_** (Scheme 38(i_2_)) or **I_4_**.

**Scheme 39:**
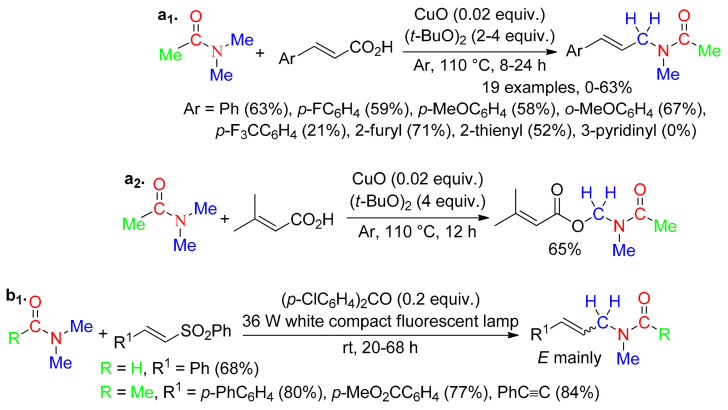

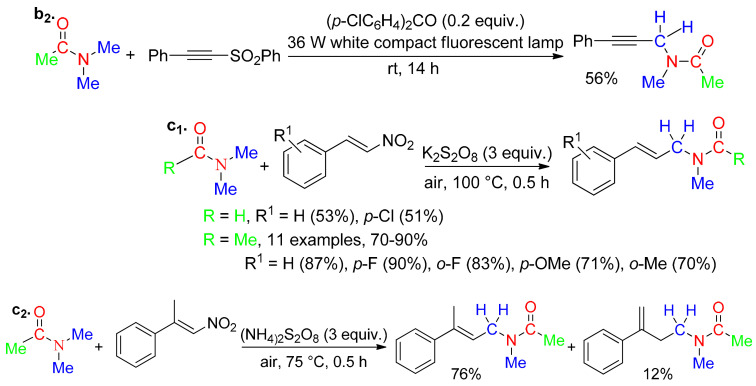
Cross-coupling of α,β-unsaturated acids, vinyl sulfones, ((phenylethynyl)sulfonyl)benzene and β-nitrostyrenes.

The CuO-catalyzed reaction of cinnamic acids with DMAc and di-*tert*-butyl peroxide led to decarboxylative alkenylation giving the corresponding *N*-cinnamyl-*N*-methylacetamides (Scheme 39(a_1_)) while 3-methylbut-2-enoic acid afforded (*N*-methylacetamido)methyl 3-methylbut-2-enoate (Scheme 39(a_2_)) [126]. Cross-coupling with the elimination of the sulfonyl or nitro group was also observed from the reaction of vinylsulfones, ((phenylethynyl)sulfonyl)benzene and β-nitrostyrenes using DM in the presence of either a diaryl ketone under visible-light irradiation (Scheme 39b) [128] or a peroxydisulfate (Scheme 39c) [129].

**Scheme 40:**
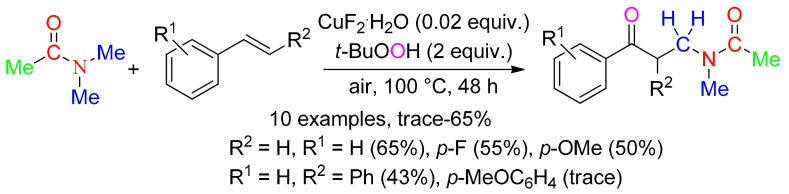
Oxyalkylation of styrenes.

Treatment at 100 °C of styrene with CuF_2_ catalyst and *t*-BuOOH led to a complicated mixture in DMF while effective production of *N*-methyl-*N*-(3-oxo-3-phenylpropyl)acetamide arose in DMAc (Scheme 40) [126]. Various vinylarenes undergo such an oxyalkylation (Scheme 40).

The above reactions involve radical intermediates **I_9_** and/or **I_4_**.

**Scheme 41:**
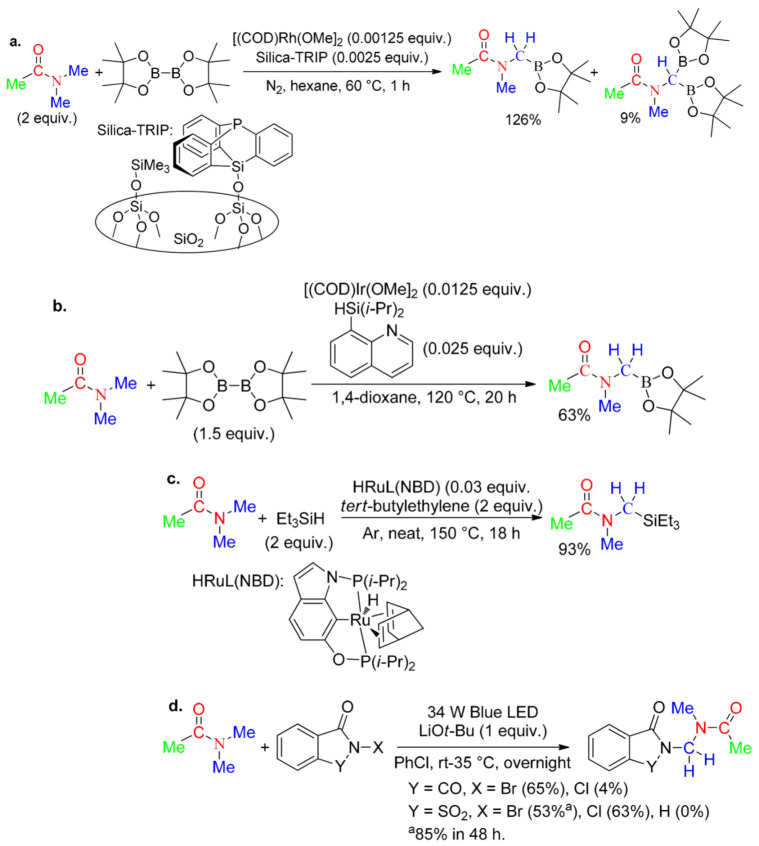
Borylation, silylation, and amidation of N-adjacent C–H bond of DMAc.

Borylation [130,131,132], silylation [133], and amidation [134] of N-adjacent C–H bond of DMAc have been achieved with bis(pinacolato)diboron and Rh or Ir catalysis (Scheme 41a,b), triethylsilyl hydride, Ru catalysis, and *tert*-butylethylene as the hydrogen acceptor (Scheme 41c), and *N*-haloimides under blue light irradiation (Scheme 41d). The transition-metal catalysis could involve the C(sp^3^)−H bond oxidative addition to the metal center [135,136] while the photochemical conditions promote the formation of radical **I_9_** [134].

## 16. RC-ONMe_2_ Fragment

**Scheme 42:**
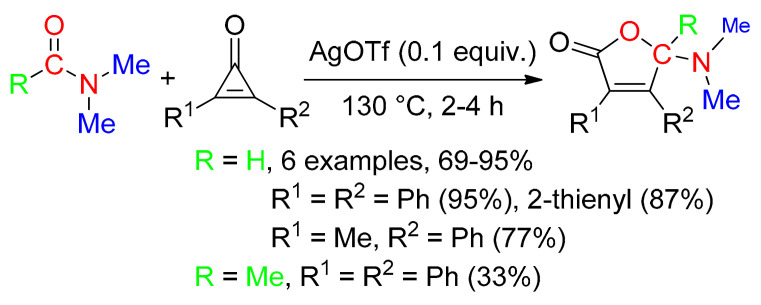
[3 + 2] Annulation.

Under AgOTf catalysis at 130 °C in DM, cyclopropenones underwent ring opening producing 5-amino-2-furanones (Scheme 42) [137]. Matsuda and co-workers proposed a reaction arising from the addition of the oxygen atom of DM to the Ag-coordinated carbonyl group of the cyclopropenone.

## 17. H_1,2_CC=ONMe_2_ Fragment, or H and H_1,2_CC=ONMe_2_ Fragments

**Scheme 43:**
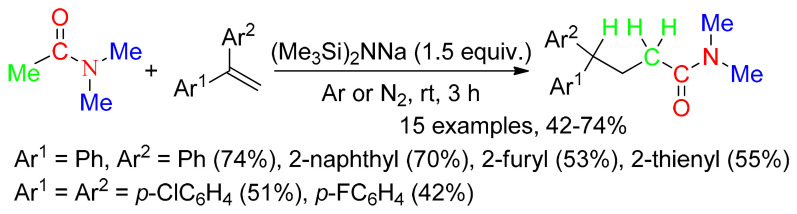
Base-promoted addition of DMAc.

*N*,*N*-dimethyl-4,4-diarylbutanamides have been synthetized at room temperature from base-mediated addition of DMAc on 1,1-diarylethylenes (Scheme 43) [138]. According to a previous report of Kobayashi’s team [139], the anionic intermediate produced from the addition of enolate **I_10_** to the substrate is protonated with an H of the Me moiety of DMAc.

**Scheme 44:**
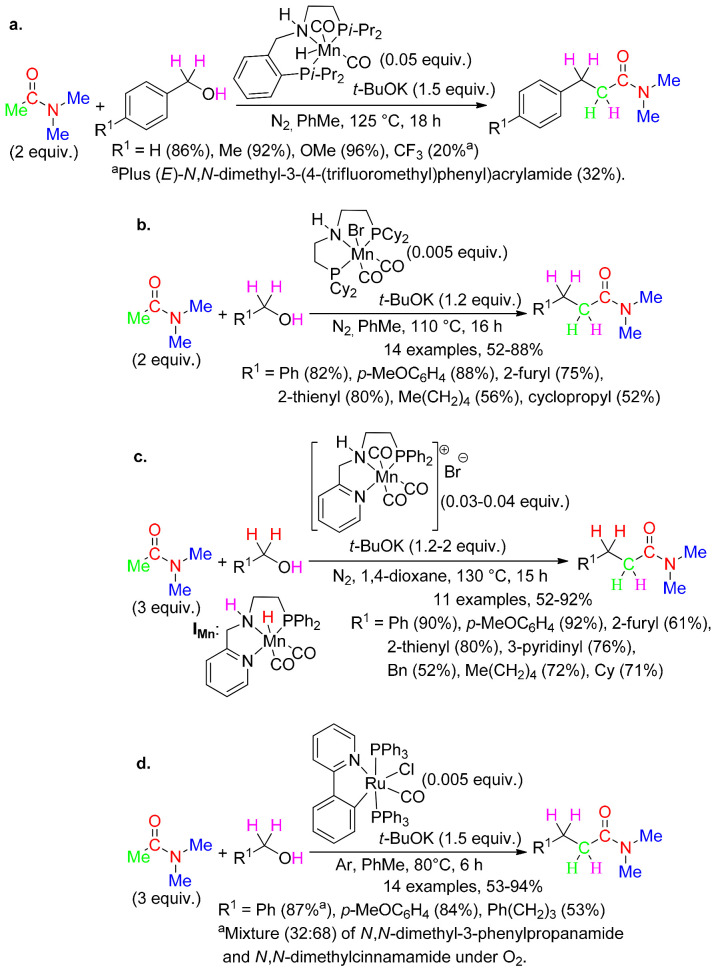

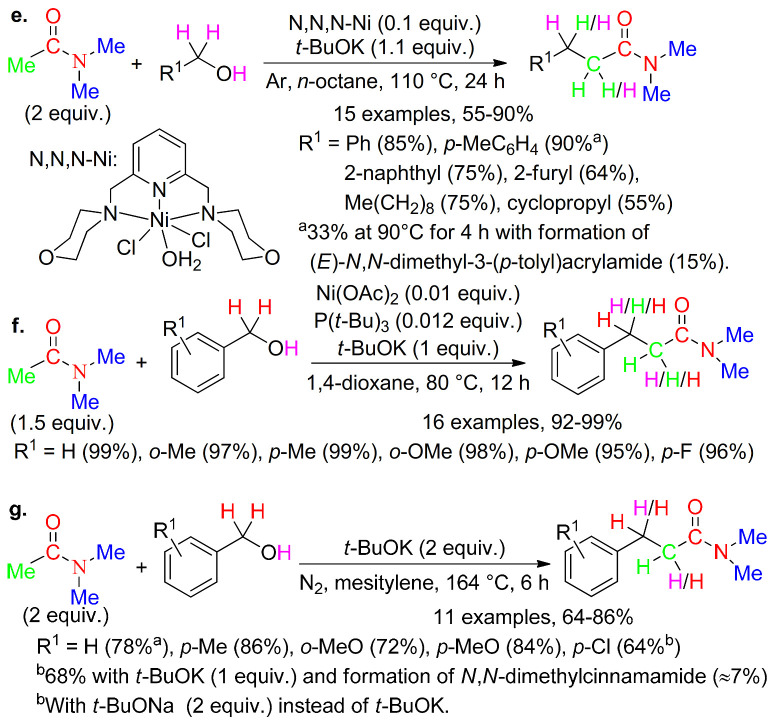
C-Alkylation of DMAc.

Mn, Rh, or Ni catalyst associated with *t*-BuOK performed effective C-alkylation of DMAc with primary alcohols (Scheme 44a–f). Hydrogen transfers are involved but plausible hydrogen exchange between alcohol and DMAc led to uncertainty about the hydrogen origin in the final product. The reactions occur via transition-metal-catalyzed oxidation of the alcohol (R’CH_2_OH) followed by base-mediated condensation with DMAc leading to the corresponding α,β-unsaturated amide—R’CH=CHCO(NMe_2_)—which was sometimes isolated as a by-product (Scheme 44a,d,e). According to the teams of Milstein [140] and Gupta and Balaraman [141], hydrogenation of the latter with H_2_ formed from alcohol oxidation provides the final product (Scheme 44a,b), whereas experiments with PhCD_2_OH led the teams of Rueping and El-Sepelgy to assume that the Mn-catalyzed alcohol oxidation produced the hydrogenated species **I_Mn_** [142]. Subsequent insertion of the C=C bond into the Mn–H bond followed by H transfer would provide the product (Scheme 44c). According to Chen’s team (Scheme 44d), the hydridorhodium species issued from Rh-catalyzed dehydrogenation of the alcohol adds to R’CH=CHCO(NMe_2_) affording an oxo-π-allylrhodium complex [143]. The reaction of the latter with R’CH_2_OH would deliver the product and the Rh alcoholate RhOCH_2_OR’ which would be the active catalytic species. Madhu, Balaraman, and their co-workers (Scheme 44e) performed a deuterium labeling experiment with *p*-ClC_6_H_4_CD_2_OD which led to a 16:36:48 mixture of D_0_, D_1_, and D_2_ 3-(*p*-chlorophenyl)-*N*,*N*-dimethylpropanamide [144]. The formation of the D_0_ product “is in agreement with the microreversibility of the initial alcohol dehydrogenation process” [144]. Yang, Zhou, Tang, and their co-workers (Scheme 44f) carried out labeling experiments with PhCD_2_OH and *t*-BuOD as an additive, but to ascribe the origin of hydrogens in α- and β-positions was also tedious [145]. It seems remarkable that the above Ni-catalyzed conditions led to the alcohol oxidation rather than to the C–N bond cleavage [33] of DMAc.

Madsen and Azizi disclosed a transition-metal-free C-alkylation of DMAc with benzylic alcohols, through a reaction mediated by *t*-BuOK or *t*-BuONa [146]. Use of 2 equiv. of the base provided the saturated amide in fair yields while lower amounts led to a mixture of the saturated and unsaturated amides (Scheme 44g). No reaction occurred with aliphatic alcohols such as hexan-1-ol and heptan-1-ol. According to the authors, the reaction occurs thanks to the dual role—base and radical initiator—of both bases (that differs from an above hypothesis, see Section 14 [95]) which initiates the formation of radical anion ArCH^∙^O^⊖^ from ArCH_2_OH. A subsequent radical chain pathway involving DMAc affords ArCH=CHCO(NMe_2_) and **I_8_**. Single-electron transfer from **I_8_** to the unsaturated amide followed by reaction with ArCH_2_OH provides the product and regenerates ArCH^∙^O^⊖^. Experiments with PhCD_2_OH did not allow to propose hydrogen distribution more accurately than the one shown in Scheme 44g.

**Scheme 45:**
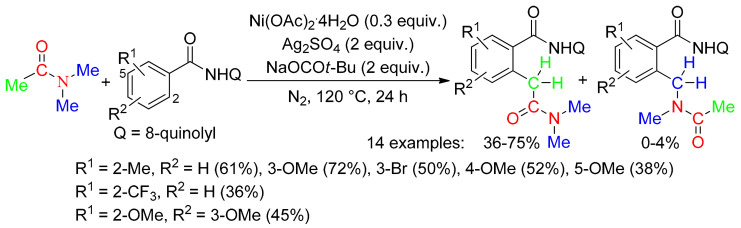
C(sp^2^)−H/C(sp^3^)−H rather than C(sp^2^)−H/C(sp^3^)−H cross-coupling.

The Ni^II^-catalyzed reaction of *N*-(quinolin-8-yl)benzamides with DMAc using Ag_2_SO_4_ and NaOCO*t*-Bu (Scheme 45) instead of (*t*-BuO)_2_ (Scheme 38(g_1_)) favored the formation of 2-(2-(dimethylamino)-2-oxoethyl)-*N*-(quinolin-8-yl)benzamides rather than that of 2-((*N*-methylacetamido)methyl)-*N*-(quinolin-8-yl) [125]. The reactions disclosed in Scheme 38(g_1_) and Scheme 45 differ strongly from that previously reported under oxygen and Ni/Cu catalysis which provided 2-(quinolin-8-yl)isoindoline-1,3-diones via carbonylation using DM as the carbon source of carbonyl group [2,3,147].

**Scheme 46:**
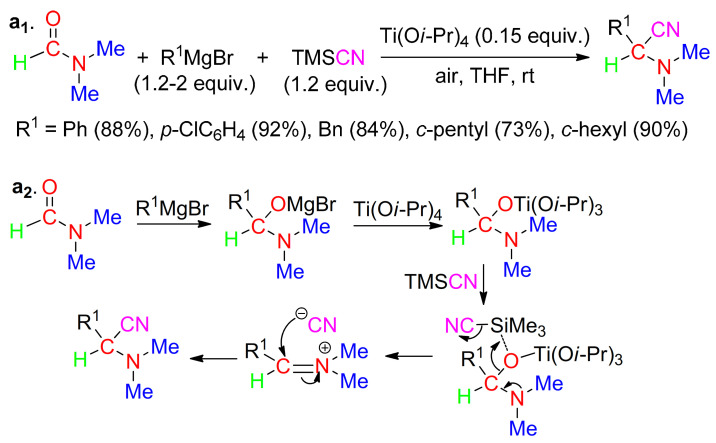
C-Alkylative cyanation of DMF.

Concomitant addition of Grignard reagents and TMSCN to DMF leading to α-amino nitriles was promoted with Ti(O*i*-Pr)_4_ catalysis (Scheme 46(a_1_)) through, according to Lannou/Sorin’s team, the addition of the Grignard reagent, transmetallation followed by reaction with TMSCN as depicted in Scheme 46(a_2_) [148].

## 18. RC and O Fragments

**Scheme 47:**
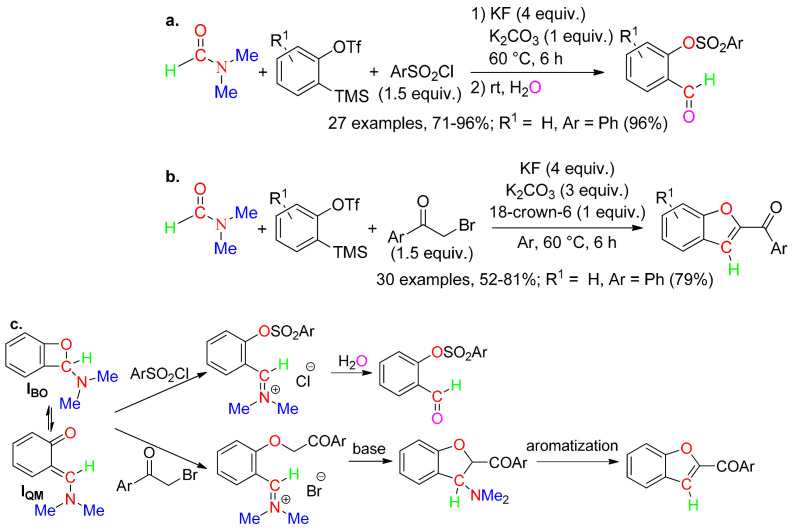
[2 + 2] Cycloadditions.

Various reactions implicating the insertion of arynes into the N–C or C=O bonds of amides have been reported [7]. In the presence of both KF and K_2_CO_3_, 2-(trimethylsilyl)phenyl trifluoromethanesulfonate reacts with DMF and either *p*-toluenesulfonyl chloride or 2-bromoacetophenone to provide 2-formylphenyl benzenesulfonate (Scheme 47a) [149] or benzofuran-2-yl(phenyl)methanone (Scheme 47b) [150], respectively. Both reactions involve benzoxetene **I_BO_** or *ortho*-quinone methide **I_QM_** obtained via KF-mediated formation of benzyne, and subsequent insertion into the C=O bond of DMF (Scheme 47c). The addition of the sulfonate followed by aqueous work-up affords 2-formylphenyl benzenesulfonate. The bicyclic compound is produced from addition to bromoacetophenone followed by base-mediated cyclization and aromatization.

## 19. HCO and C-O Fragments

**Scheme 48:**
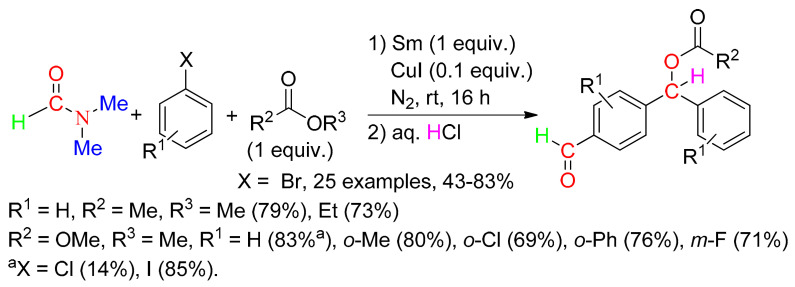
Diarylmethanol skeletons via three-component reactions.

Recently, the Qi/Liu team proposed a Sm/CuI-mediated reaction between DMF, aryl halides, and esters or diesters leading to functionalized diaryl methanols (Scheme 48) [151]. The mechanism is unclear. Formylation of ArX following by some coupling between two molecules of ArCHO and the ester could be involved [151].

## 20. H and NMe_2_ Fragments

**Scheme 49:**
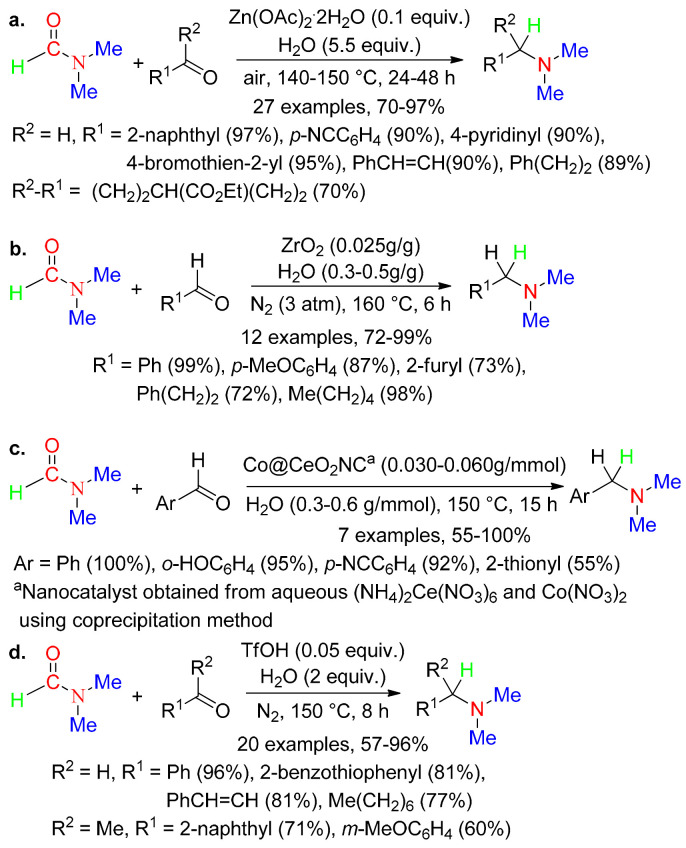
Reductive amination of aldehydes and ketones.

The Leuckart-type reaction between wet DMF and aldehydes or ketones arose at 140–160 °C under catalysis with various Lewis [152,153,154] or Brønsted [93] acids (Scheme 49). The use of DCON(CD_3_)_2_ led to the *d_7_*-reductive amination product [93,152]. The acidic conditions cause the formation of HCOOH and HNMe_2_ from HCONMe_2_ and H_2_O. The subsequent condensation of the primary amine with the substrate (R^1^R^2^C=O) generates the iminium cation R^1^R^2^C=N^⊕^Me_2_ which undergoes reduction with HCOOH leading to R^1^R^2^CH(NMe_2_).

## 21. CH_1,2_ and NMe_2_ Fragments

**Scheme 50:**
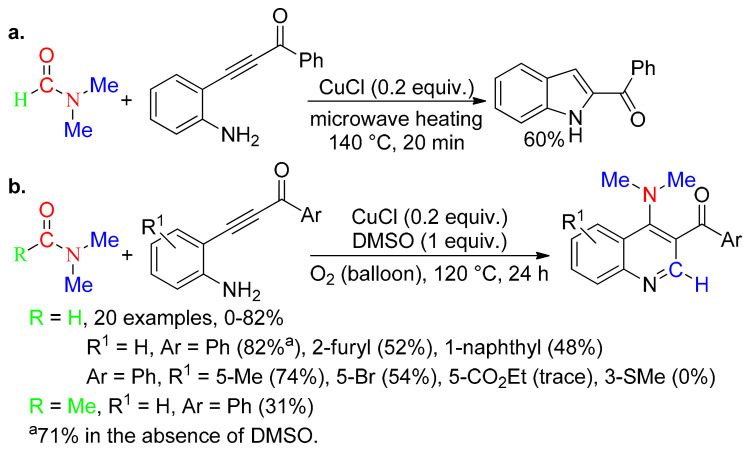

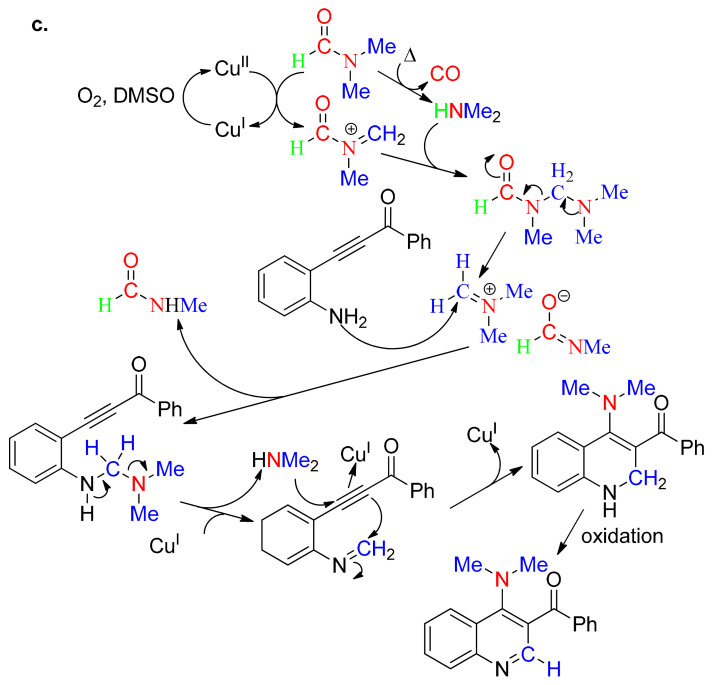
Hydroamination or addition/cyclization/oxidation sequence.

While Marinelli’s team isolated (1*H*-indol-2-yl)(phenyl)methanone in 60% yield from microwave heating (140 °C) in DMF of *o*-phenylethynyl aniline in the presence of 0.2 equiv. of CuCl (Scheme 50a) [155], Lin and co-workers subsequently obtained (4-(dimethylamino)quinolin-3-yl)(phenyl)methanone in 71% yield from the reaction at 120 °C of the same substrate, in the same solvent with the same amount of CuCl, but under oxygen atmosphere, the yield increased to 82% with DMSO as the additive (Scheme 50b) [156]. As Marinelli’s report was not cited by Lin’s team, no explanation of the reactivity difference was provided. The discrepancy between the two reports is plausibly due to the oxidation medium of the second paper. According to DFT calculations reported in the first paper, activation of the triple bond by coordination to a Cu^I^(DMF) complex promotes intermolecular hydroamination, with preservation of the oxidation state of the catalyst [155]. Lin and co-workers assumed that oxygen oxidizes Cu^I^ into Cu^II^ [156], a reaction probably promoted by DMSO [157,158]. This redox system is associated with the thermal decomposition of DMF, a decisive step of the proposed mechanism, which agrees with labeling experiments using DCON(CD_3_)_2_ and H^13^CON(CH_3_)_2_. Scheme 50c slightly differs from that proposed by the authors. The pivotal role of the experimental conditions on the Cu-catalyzed reaction of *o*-phenylethynyl aniline has to be highlighted. Indeed, Lin’s team previously reported the production of 2-phenyl-1*H*-indole-3-carbaldehyde from *o*-phenylethynyl aniline under Cu(OCOCF_3_)_2_^.^xH_2_O catalysis and O_2_ atmosphere in DMF at 120 °C (Scheme 3(b_1_)) [20], that is under experimental conditions very close to those they subsequently used (Scheme 50b) [156] but the striking reactivity difference was again neither explained nor pointed out by the authors.

**Scheme 51:**
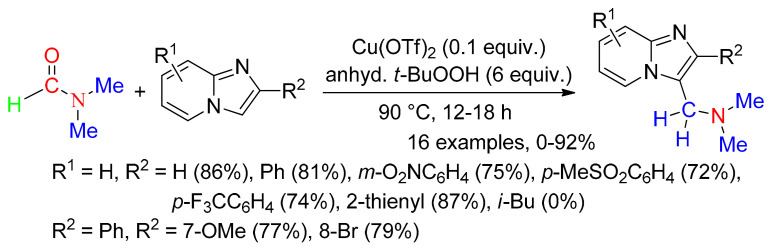
Aminomethylation.

Hajira’s team disclosed the Cu^II^-catalyzed aminomethylation of imidazopyridines with DMF and *t*-BuOOH (Scheme 51) [159]. According to the authors, the reaction arises via addition to the substrate of H_2_C=N^⊕^Me_2_, formed as depicted in Scheme 50c but using *t*-BuOOH as the oxidant.

## 22. CH and N Fragments

**Scheme 52:**
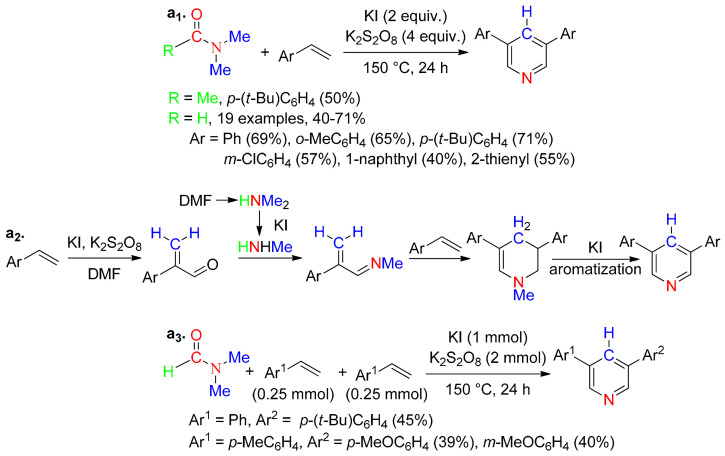
Annulation reaction between styrenes and DM.

Recently, the Liu and Guo team disclosed the synthesis of symmetrical 3,5-diarylpyridines from the KI/K_2_S_2_O_8_-mediated reaction of styrenes with DM, especially DMF (Scheme 52(a_1_)) [160]. In contrast to the examples shown in Scheme 5(a_2_), DM provided both the nitrogen atom and the methine fragment. The α,β-unsaturated aldehyde depicted in Scheme 52(a_2_) has been identified as intermediate. After the formation of the corresponding aldimine, [4 + 2] cycloaddition with styrene is followed by KI-mediated N-Me bond cleavage and aromatization. Unsymmetrical 3,5-diarylpyridines have been isolated using 1:1 mixtures of two different styrenes (Scheme 52(a_3_)).

## 23. Conclusions

The present review shows that various new procedures have continued to be disclosed over the last years using DMF and DMAc as sources of building blocks for the synthesis of an array of organic compounds. For processes involving atom(s) of the Me_2_NCO moiety of DM, efficiency and selectivity are usually higher with DMF than with DMAc. In contrast, the latter is generally the best for carbamoylation reactions. Intensive mechanism studies were sometimes required to determine the atom origin as exemplified for a rather banal reaction such as the formylation with HCON(CH_3_)_2_, the formyl moiety coming from COH, CH and O of H_2_O, or CH and O of O_2_. Some uncertainty nevertheless remains for a few reactions.

Numerous procedures above documented have been used for syntheses with other amides as sources of building blocks; others could also be efficient. Another remark concern the alarm to the potential safety hazards associated with using DM in particular chemical conditions [161,162] and the toxicity of these solvents [163].
